# Co-Occurring Diseases and Mortality in Patients With Chronic Heart Disease, Modeling Their Dynamically Expanding Disease Portfolios: Nationwide Register Study

**DOI:** 10.2196/57749

**Published:** 2025-04-25

**Authors:** Nikolaj Normann Holm, Anne Frølich, Helena Dominguez, Kim Peder Dalhoff, Helle Gybel Juul-Larsen, Ove Andersen, Anders Stockmarr

**Affiliations:** 1 Department of Applied Mathematics and Computer Science Technical University of Denmark Kgs. Lyngby Denmark; 2 Innovation and Research Centre for Multimorbidity Slagelse Hospital Slagelse Denmark; 3 Department of Public Health University of Copenhagen Copenhagen Denmark; 4 Department of Biomedical Sciences University of Copenhagen Copenhagen Denmark; 5 Department of Clinical Pharmacology Bispebjerg and Frederiksberg University Hospital Copenhagen Denmark; 6 Department of Clinical Medicine University of Copenhagen Copenhagen Denmark; 7 Department of Clinical Research Copenhagen University Hospital Amager and Hvidovre Hvidovre Denmark; 8 Emergency Department Copenhagen University Hospital Amager and Hvidovre Hvidovre Denmark

**Keywords:** survival analysis, interaction effects, chronic heart disease, multimorbidity, time-varying covariates

## Abstract

**Background:**

Medical advances in managing patients with chronic heart disease (HD) permit the co-occurrence of other chronic diseases due to increased longevity, causing them to become multimorbid. Previous research on the effect of co-occurring diseases on mortality among patients with HD often considers disease counts or clusters at HD diagnosis, overlooking the dynamics of patients’ disease portfolios over time, where new chronic diseases are diagnosed before death. Furthermore, these studies do not consider interactions among diseases and between diseases, biological and socioeconomic variables, which are essential for addressing health disparities among patients with HD. Therefore, a mapping of the effect of combinations of these co-occurring diseases on mortality among patients with HD considering such interactions in a dynamic setting is warranted.

**Objective:**

This study aimed to examine the effect of the co-occurring diseases of patients with HD on mortality, modeling their dynamically expanding chronic disease portfolios while identifying interactions between the co-occurring diseases, socioeconomic and biological variables.

**Methods:**

This study used data from the national Danish registries and algorithmic diagnoses of 15 chronic diseases to obtain a study population of all 766,596 adult patients with HD in Denmark from January 1, 1995, to December 31, 2015. The time from HD diagnosis until death was modeled using an extended Cox model involving chronic diseases and their interactions as time-varying covariates. We identified interactions between co-occurring diseases, socioeconomic and biological variables in a data-driven manner using a hierarchical forward-backward selection procedure and stability selection. We mapped the impact on mortality of (1) the most common disease portfolios, (2) the disease portfolios subject to the highest level of interaction, and (3) the most severe disease portfolios. Estimates from interaction-based models were compared to an additive model.

**Results:**

Cancer had the highest impact on mortality (hazard ratio=6.72 for male individuals and 7.59 for female individuals). Excluding cancer revealed schizophrenia and dementia as those with the highest mortality impact (top 5 hazard ratios in the 11.72-13.37 range for male individuals and 13.86-16.65 for female individuals for combinations of 4 diseases). The additive model underestimated the effects up to a factor of 1.4 compared to the interaction model. Stroke, osteoporosis, chronic obstructive pulmonary disease, dementia, and depression were identified as chronic diseases involved in the most complex interactions, which were of the fifth order.

**Conclusions:**

The findings of this study emphasize the importance of identifying and modeling disease interactions to gain a comprehensive understanding of mortality risk in patients with HD. This study illustrated that complex interactions are widespread among the co-occurring chronic diseases of patients with HD. Failing to account for these interactions can lead to an oversimplified attribution of risk to individual diseases, which may, in cases of multiple co-occurring diseases, result in an underestimation of mortality risk.

## Introduction

### Background

Driven by the advancements in diagnostic tools and medical treatments, the mortality of patients with chronic heart disease (HD) has decreased considerably [1]. However, with a prolonged life span comes a risk of developing additional chronic diseases and complications to their HD [2], causing them to become multimorbid [3]. Multimorbidity is highly prevalent among patients with HD [2,4,5], and the increasing disease burden may modify time to death [6].

Recent research has identified the most prevalent comorbidities in patients with HD and how they affect mortality and other adverse health-related outcomes [5,7-9]. However, only a few studies have considered the effect of several diseases in the same person. Among these studies, there is a large variety in which diagnoses are considered and which statistical methods are applied. The studies that consider multimorbidity either restrict their analyses to a subset of diagnosis combinations [7] or group diagnoses into multimorbidity clusters at baseline before analyzing the effects of the extracted clusters [5]. Despite modeling disease interactions, these kinds of analyses fail to capture the crucial dynamics in the HD disease trajectories, where additional diseases are cumulatively diagnosed before death [10], causing an augmented risk profile for the patient. As the chronology of disease onset has been associated with a change in mortality among common diagnoses [11], it is thus essential to consider this dynamic development when analyzing effects. Due to the high prevalence of multimorbidity among patients with HD, the unique combination of chronic diseases that a patient has at any given time—referred to as the *disease portfolio*—is not static. Instead, it evolves over the observation period as new chronic diseases develop. This dynamic expansion reflects the progressive accumulation of chronic diseases in an individual following their HD diagnosis until death. As only a few studies consider these dynamics, there is a need for a thorough, large-scale study of the impact of disease interactions on mortality, modeling such a dynamic expansion of the patients’ disease portfolios. Such an investigation would enable obtaining a deeper understanding of how the complexity of disease progression in patients with HD affects mortality over time.

The significance of understanding the effects of the emergence of multimorbidity over an individual’s life span has previously been highlighted [3,12,13]. However, rather than treating multimorbidity as a singular risk factor, we took a more nuanced approach by dissecting the effects of multimorbidity based on the diseases appearing in the disease portfolio, recognizing that each combination of chronic diseases can affect mortality differently. Furthermore, as many chronic diseases have similar biological and socioeconomic risk factors, knowledge of the interplay between the impact of these is essential and can be used for possible preventive interventions and the development of guidelines for relevant coexisting diseases [14,15]. For instance, consider a disease portfolio comprising HD and osteoporosis. The impact on the mortality hazard rate may vary between men and women. Expanding on this example, the effect of socioeconomic position may differ depending on both sex and the presence of osteoporosis in the portfolio. These variations in effects represent interactions in modeling terms. As such, identifying and emphasizing interactions between chronic diseases and demographic factors can shed new light on the impact of pathophysiological pathways on mortality.

### Objectives

**Figure 1:**
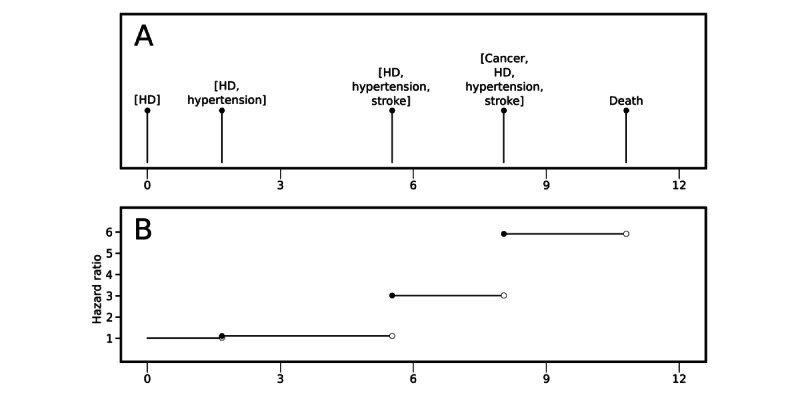
Example of how the statistical model works. (A) Illustration of an event sequence in which a hypothetical patient with heart disease (HD) receives the diagnosis of HD at time 0 and, subsequently, the hypertension, stroke, and cancer diagnoses at different times (measured in years following HD diagnosis) before death. (B) The corresponding longitudinal development of the hazard ratio of the patient relative to a theoretical patient who only has HD and is not multimorbid.

This large-scale study is based on data from the total adult Danish population recorded in nationwide primary and secondary health care registries, including medical diagnoses, medications, educational attainment level, and health care use. We used an extended Cox model with time-varying covariates to model time until death for individuals diagnosed with HD considering their dynamically expanding disease portfolios. In our model, the hazard ratio (HR) of a disease portfolio is constant. In contrast, the HR of an individual changes dynamically when the individual obtains a new portfolio by developing a new chronic disease (Figure 1).

We conducted a model and data-driven selection of interaction effects. Subsequently, we studied the impact on time to death according to the (1) most frequently occurring disease portfolios, (2) most complex disease portfolios in terms of order of interactions, and (3) disease portfolios with the highest hazards relative to only HD.

We recognize the inherent complexity in interpreting interaction effects, especially in the case of higher-order interactions involving multiple factors. However, to emphasize the importance of modeling interaction effects, we also present a comparative analysis of effect estimates for disease portfolios, contrasting our interaction model with a simpler model in which interactions are excluded. The differences observed in these comparisons serve to underscore the crucial role of modeling interactions in medical research.

Throughout this paper, we use a bracket notation to represent the disease portfolio of a specific patient with HD. For example, a patient with HD, diabetes, and hypertension is denoted by the portfolio [diabetes, hypertension]. If the patient with HD also has high cholesterol, their disease portfolio is [diabetes, high cholesterol, hypertension]. As all individuals in the study population had HD, we use the term *disease portfolio* without mentioning the coexisting HD diagnosis in the notation. We use the terms *dyads*, *triads*, *tetrads*, and *pentads* to describe disease portfolios of size 2, 3, 4, and 5, respectively, with size being the number of chronic diseases in the portfolio including HD.

## Methods

### Data Foundation

All children born in Denmark or any new residents are, by law, required to obtain a unique personal identification number, which is stored in the Danish Civil Registration System [16]. The personal identification number can link information from any additional Danish register at an individual level subject to General Data Protection Regulation restrictions [17]. Information about chronic disease diagnoses was based on diagnostic algorithms initially developed by the Research Center for Prevention and Health at Glostrup University Hospital [18]. These algorithms cover 15 diagnoses based on their clinical relevance that have been previously used in national reports of chronic disease diagnoses in Denmark [19,20]. Moreover, previous work with these diagnoses has shown prevalence results comparable to those of other European studies [21]. The algorithmic diagnoses are based on data recorded in 4 registries: the Danish National Patient Register [22], the Danish Psychiatric Central Research Register [23], the Danish National Prescription Registry [24], and the Danish National Health Service Register [25]. Therefore, a particular diagnosis can be given at a particular time (with temporal granularity of days) based on criteria for hospitalization diagnoses, medication, or repeated use of specific health services. As such, a single diagnosis corresponds to 1 disease and represents multiple Anatomical Therapeutic Chemical or *ICD-10* (*International Statistical Classification of Diseases, 10th Revision*) codes with similar treatments and organization of health care. Thus, the diagnosis time stamps considered in this study are diagnostic time stamps and should not be regarded as time stamps for disease onset. In addition to the registries used for diagnostic time stamps, we used the Danish Population Education Register [26] and the Danish Register of Causes of Death [27] for information on educational attainment and death.

### Study Design and Population

Using our data foundation from the Danish registries, we obtained a study population of individuals diagnosed with HD covering the entire Danish adult population (aged ≥18 years) at some point during the observation period from January 1, 1995, to December 31, 2015, which had been previously analyzed [28]. These people were followed up on, and data associated with visits to outpatient clinics, hospital stays, primary sector health services, and prescriptions were collected for each person throughout the observation period. To define the study population, we applied algorithmic diagnoses (detailed in Multimedia Appendix 1) to identify individuals diagnosed with HD while determining diagnostic time stamps for 14 additional selected chronic diseases [21]. Thus, our inclusion criterion was broad, encompassing all Danish adults (aged ≥18 years) who received an algorithmic diagnosis of HD during the study period. No additional exclusion criteria were applied. Our outcome was time until death of any cause after the HD diagnosis.

### Statistical Analysis

The prevalence of each of the chronic diseases was calculated at the time of HD diagnosis across all patients in the population. Similarly, we calculated prevalences of the diseases throughout the observation period by considering whether the condition had occurred at all among the patients with HD.

The data were analyzed within a survival analysis framework, with years following HD diagnosis as the time variable and an event defined as all-cause mortality. As such, we denoted the HD diagnosis time as *t*=0 and aligned our timescale accordingly, meaning that time *t*=0 corresponds to potentially different age times and calendar times for distinct individuals. In addition, individuals lost to follow-up due to emigration or reaching the end of the observation period were censored at these times.

The time-varying information on individual diagnoses; information on sex (male or female), age, educational attainment level (none, short, medium, long, missing, and missing before 1920); and calendar time were included as explanatory variables in the analysis (refer to tables 1/2 in the study by Holm et al [28]). We used an extended Cox model to estimate the effect of these explanatory variables on mortality, allowing for the inclusion of time-varying covariates. We classified our variables into primary and intrinsic categories [29]. Primary variables, such as the time-varying diagnosis indicators, cover variables of paramount interest. Intrinsic variables define the study individuals (ie, the variables sex, age, educational attainment level, and calendar time). Interactions both between and within each group of variables were considered. The numerical variables were mean centered before analysis.

As the development of additional diagnoses is a continuous process, the primary variables were allowed to change over time. These variables were piecewise constant in time, being 0 when the diagnosis was not present and 1 when obtained and onward in time. As the registries continuously cover clinical events for all individuals over the observation period, these diagnosis variables update at individual-specific time points dictated by the (sequence of) events that trigger the algorithmic diagnosis (Multimedia Appendix 1). An example of a potential sequence of diagnoses is showcased in Figure 1.

In the extended Cox proportional hazard model [30], the hazard *h_i_* for the *i*th individual at time *t* is given by the following equation:



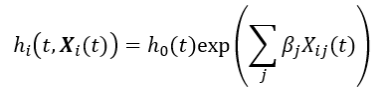



**(1)**

In this equation, *h*_0_(*t*) is the unspecified baseline hazard function for a male individual with no education without any diagnoses except HD. *X_ij_*(*t*) denotes the variable *j* for individual *i* (with ***X****_i_*(*t*) denoting a vector of all variables) at time *t*, with *i*=1,...,*n*. The β*_j_* are the effect parameters. Due to *h*_0_(*t*) being unspecified, these parameters are linked to the relative mortality hazard rate of a variable as opposed to the absolute risk. Equation 1 assumes that variables have proportionate effects on the hazard function over time. We assessed this assumption for each variable by examining Schoenfeld residuals [31]. In addition, as the effect parameters β*_j_* do not depend on time, the hazard rate associated with a particular combination of explanatory variables was assumed to be the same across all time points.

To analyze the data, the following software was used: R (version 4.2.2; R Foundation for Statistical Computing), with the packages *survival* (version 3.5-5), *lava* (version 1.7.1), *glmnet* (version 4.1-6), and *multcomp* (version. 1_4-20).

### Selection of Variables and Interactions

It is essential to account for diagnosis interactions as such parameters serve to model the entire effect of disease portfolios associated with mortality. Possible omitted interaction effects from a model in which a significant interaction exists can result in a misrepresentation of the relationship between the variables and the time until death. It may also lead to bias in parameter estimation [32,33].

A common way to perform variable selection is a backward selection approach starting from a full model considering all possible interactions, reducing it to a model that best explains the observed data. However, such an approach was not computationally feasible as we are in a big data setting with numerous observations and countless potential variable interactions. Instead, we considered 2 variations of a forward-backward selection procedure to discover disease interactions. As a sensitivity analysis, we also performed variable selection using the stability selection methodology [34] with the regularization-based least absolute shrinkage and selection operator (LASSO) [35] approach.

In addition to the models including interaction effects, a model solely consisting of the primary and intrinsic variables’ main effects (and squared and cubic terms) was estimated for reference.

We considered *k*-way interactions iteratively for *k*=2,..., *M*, with *M* being a predetermined upper limit. The selection procedure starts from an initial model including all main effects and works in the following way for each value of *k*:

- Generate *n_c_* candidate variable additions obtained from the current model by adding a single *k*-way interaction to an already existing (*k* – 1)–way interaction, also adding necessary lower hierarchical terms.
- Repeat until there are no candidate models below the cutoff: (1) estimate each of the candidate models obtained from adding any of the *n_c_* variables not already added to the current model and compare with the current model using a likelihood ratio test and (2) select the candidate model with the lowest *P* value below the cutoff α/*n_c_* as the current model.
- Clean up potentially masked significances in the *k*-way selection path through backward selection using a test level of α.

The selection algorithm runs either until *M*-way interactions are included or until no *k*-way interactions are selected in the *k*th iteration. In the forward step of the selection algorithm, a Bonferroni-adjusted cutoff is used to minimize the risk of false discoveries as each variable addition is potentially tested for inclusion *n_c_* times. We note that all considered models are hierarchical, meaning that, if a model contains a 5-way interaction among 5 variables, it also contains all possible 4-, 3-, and 2-way interactions among those variables.

Due to the allowance of *any k*-way interaction between and among the primary and intrinsic variables, a possibly large number of candidate models were included for each value of *k*. Because of this, the selection forward step was relaxed such that the candidate model *P* values were ordered from lowest to highest after the first estimation for each value of *k*. In the following estimations, variable additions were checked in this order, immediately adding any interactions below the cutoff while discarding insignificant terms. Before backward selection, any discarded terms were included again through forward selection. To introduce conservatism, all variable selections were performed with α=.001. The resulting model with all selected interactions was labeled as the ALL model.

In addition to the ALL model, the variable selection procedure was run without relaxation of the forward step but only considering interactions among the primary variables. We labeled this as the disease interactions only (DIO) model. Furthermore, we used a variation of the stability selection framework [34], a method for improving variable selection in high-dimensional, sparse environments. This method selects variables repeatedly chosen on subsampled data through a structure learning method such as the LASSO algorithm for the Cox model [36]. We used a selection threshold of 0.9 following the recommendation in the work by Meinshausen and Bühlmann [34]. Each subsample included 10 randomly selected variables considering all their possible interactions up to an order of 5. This caused us to consider 3400 subsamples in total. We then fit an unregularized Cox model using the stably selected terms and performed backward selection to reduce the model using all available data. The resulting model was labeled the stable model. As a sensitivity criterion, we compared detected interactions among the chronic diseases across the ALL, DIO, and stable models. The additive model only including main effects was labeled as the only main effects (OME) model.

### Selecting Disease Portfolios

Due to the many possibilities when considering combinations of the 14 co-occurring diseases, some of our presented results are based on selected disease portfolios. These selections were made based on 3 criteria: most common disease portfolios, disease portfolios subject to the highest order of disease interactions, and disease portfolios with the highest mortality impact. The main results presented in this paper are based on the ALL model. To illustrate the importance of modeling interaction effects, the effect of specific disease portfolios in the ALL model was compared to additive effects from the OME model on the log-hazard scale.

### Scenarios

As the considered diagnosis variables were subject to higher-order interactions, effects were not apparent just from the estimated parameters because the effect of a single diagnosis varied across different levels of other diagnoses and intrinsic variables. To supplement the effect of the selected disease portfolios, the absolute mortality risk over time was estimated for multiple scenarios using the estimated ALL model. We did this to illustrate the modification of the risk profile over time of an individual diagnosed with HD. Each scenario represented the risk of a hypothetical individual whose disease portfolio expands at predetermined time points following HD diagnosis. The times at which the disease portfolio expanded in the hypothetical scenarios were determined in a data-driven fashion using gamma regressions, where the time points (at which the first, second, or third expansion of the disease portfolio following HD diagnosis occurred) were regressed on the diagnoses in the sequence considered in the scenario. The scenarios were constructed for patients who received their HD diagnosis at mean age and calendar time levels.

### Ethical Considerations

In this study, we used data from the national Danish registries, which are protected by the Danish Data Protection Act, meaning that they can only be accessed after application and subsequent approval. This study did not require additional approval from the Danish Research Ethics Committees or any informed consent as it solely involved the use of national registry data, exempt under the Scientific Ethical Committees Act. Danish registry data are deidentified to protect the privacy of individuals.

## Results

### Characteristics of the Study Population

**Figure 2:**
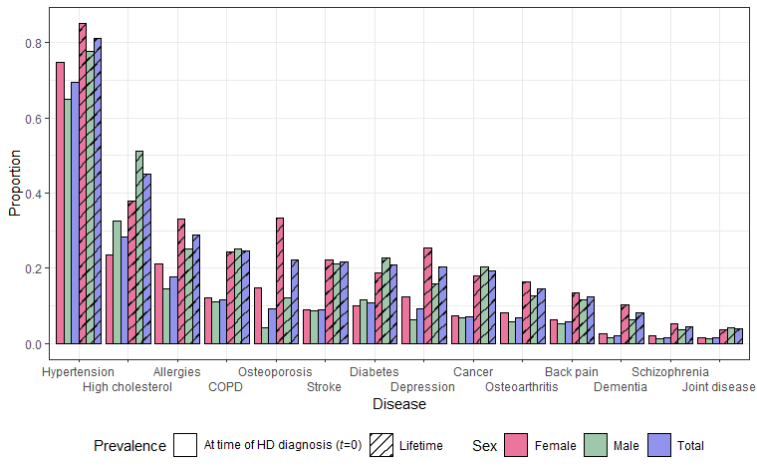
Diagnosis prevalence according to sex. Prevalence is reported at the time of heart disease (HD) diagnosis and for the entire span of the observed disease trajectories (Lifetime). COPD: chronic obstructive pulmonary disease.

A total of 766,596 individuals diagnosed with HD were included (n=406,792, 53.06% male). The mean age at the time of HD diagnosis was 67.51 (SD 13.07) years for male individuals and 73.02 (SD 13.37) years for female individuals (further baseline characteristics are available in table 2 in the work by Holm et al [28]). At the end of the observation period, 57.95% (444,233/766,596) were dead (222,112/406,792, 54.6% male and 222,121/359,804, 61.73% female). Overall, the prevalence of multimorbidity in the complete trajectories of each patient with HD was 96.88% (742,688/766,596). This was an increase compared to the multimorbidity prevalence at time *t*=0 (661,490/766,596, 86.29%). The prevalence of each of the 14 co-occurring diseases is presented in Figure 2. Overall, hypertension, high cholesterol, and allergies were among the most prevalent diseases in the HD population, with a lifetime prevalence of 81.18% (622,323/766,596), 44.94% (344,481/766,596), and 28.88% (221,385/766,596), respectively (Figure 2; Multimedia Appendix 2). Differences in prevalence by sex were large for some chronic diseases, particularly for osteoporosis and depression, commonly occurring in female individuals.

### Interactions

**Figure 3:**
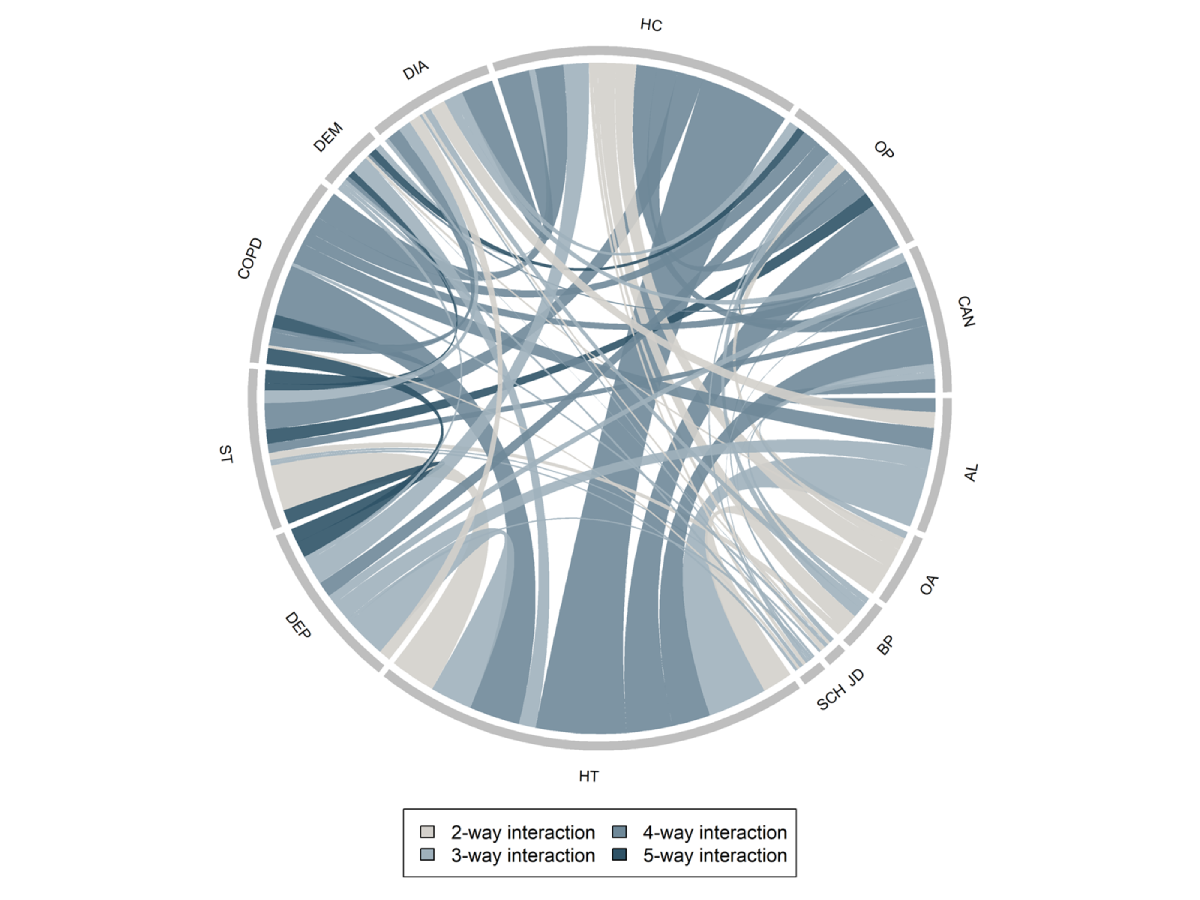
Graphical representation of disease-disease interactions in the all interactions model. A ribbon connects chronic diseases that have any significant interaction (P<.001) between them. The connection’s width corresponds to the number of individuals diagnosed with HD developing both diseases throughout the observation period. The ribbon’s color represents the highest-order interaction relationship between 2 diseases. The ribbon chart is ordered by number of connections between diseases, starting from allergies (AL) with 5 connections all the way to cancer (CAN), which interacts with all the additional diseases. BP: back pain; COPD: chronic obstructive pulmonary disease; DEM: dementia; DEP: depression; DIA: diabetes; HC: high cholesterol; HT: hypertension; JD: joint disease; OA: osteoarthritis; OP: osteoporosis; SCH: schizophrenia; ST: stroke.

Following the inclusion of 5-way interactions, the ALL model selection procedure terminated due to no 6-way interactions being selected. All the primary and intrinsic variables were present in the final model. Figure 3 illustrates statistically significant (*P*<.001) interaction relationships between chronic diseases detected in the ALL model. Connections between diseases in the ribbon chart illustrate the 2 chronic diseases appearing in an interaction, with the color depicting the complexity of the interaction (darker color represents a higher-order interaction). The figure shows all diseases interacting, with some diseases involved in more complex interactions than other chronic diseases. In total, 288 interactions were present in the final model. The interaction relationships between the considered diseases were highly diverse but dominated by cancer, which had statistically significant interactions with all other diseases. Depression, stroke, chronic obstructive pulmonary disease (COPD), dementia, and osteoporosis were involved in the most complex interactions as they were the sole diseases involved in 5-way interactions. Some of the most prevalent diseases, allergies and hypertension, were not part of these complex relationships.

The chronic disease allergies were part of 5 interaction relationships with other diseases, involving two 4-way, two 3-way, and a single 2-way interaction. Hypertension interacted with 9 other diseases, involving four 4-way, three 3-way, and two 2-way interactions. Notably, dementia and depression appeared in higher-order interactions (two 5-way interactions) despite having fewer co-occurrences in the population. Similar patterns were observed for the DIO and stable models (Multimedia Appendices 3 and 4). In both models, COPD, dementia, stroke, and depression were involved in interactions of the highest order. The DIO model included up to 5-way interactions, also featuring complex interactions involving the chronic diseases diabetes and cancer (Multimedia Appendix 3). For the stable model, only up to 4-way interactions were detected (Multimedia Appendix 4). In general, most of the interactions between diseases identified in the ALL model were also present in the DIO and stable models (Multimedia Appendix 5).

### Effects

#### Difference in Effect Estimates by Disease Portfolio Size

**Figure 4:**
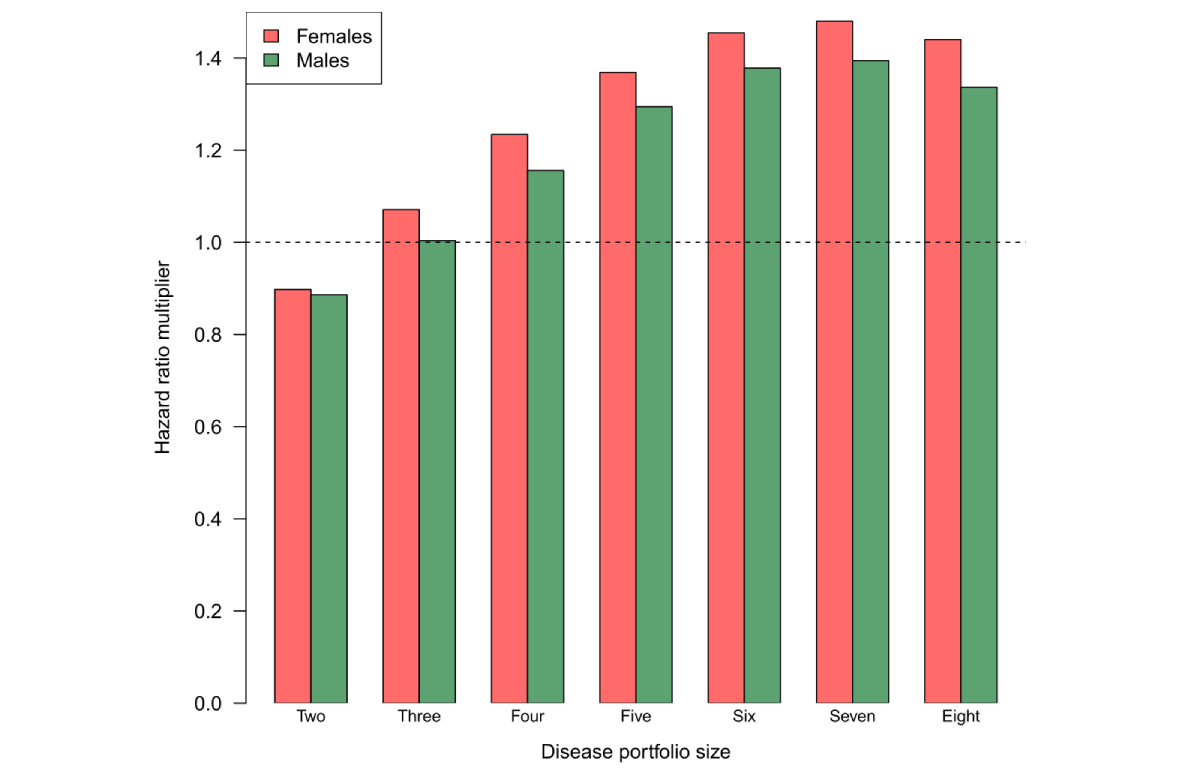
Difference in effect estimates for disease portfolios of increasing size for female and male individuals. Each bar represents a weighted average of the differences in effects between the additive only main effects (OME) model and the all interactions (ALL) model on the hazard scale exp(Inline Graphic 3). Thus, the bars indicate the average multiplier required to convert the hazard ratio (HR) from the OME model into the HR from the ALL model. The weights were determined based on the occurrence of each specific disease portfolio across the different educational attainment levels for each sex.

To evaluate how the effects of disease portfolios on time until death differed between models with and without interactions, we calculated the effect differences between the OME model and the ALL model on the log-hazard rate scale, denoted as 
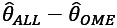
. We focused on disease portfolios ranging from 2 to 8 diseases as these accounted for 98.95% (1,671,575/1,689,297) of all disease portfolio observations of size ≥2. The effects in the ALL model for each disease portfolio were computed at mean age and calendar time levels, aggregating over combinations of both sexes and all educational attainment levels. To compute an overall estimate of the effect differences between the models for each sex, we calculated a weighted mean of the differences for each portfolio size. The weights were determined by the prevalence of individual disease portfolios across the different educational levels for each sex. In Figure 4, the aggregated differences are displayed on the hazard scale, indicating the multiplier required to convert the HR from the OME model into the HR from the ALL model. The figure illustrates substantial variations in disease portfolio effects when interactions were excluded compared to when they were included. The HR multiplier increased gradually for disease portfolios of increasing size, flattening at approximately 1.4 at disease portfolios of size 6. In general, for disease portfolios of size 2, the HRs were, on average, slightly overestimated when interactions were not modeled. However, for disease portfolios of size ≥4, the HRs were, on average, underestimated for both sexes. The underestimation also applies to female individuals with disease portfolios of size 3. In general, the HR multiplier was slightly greater for female individuals compared to male individuals across all disease portfolios.

#### Most Frequent Disease Portfolios

**Figure 5:**
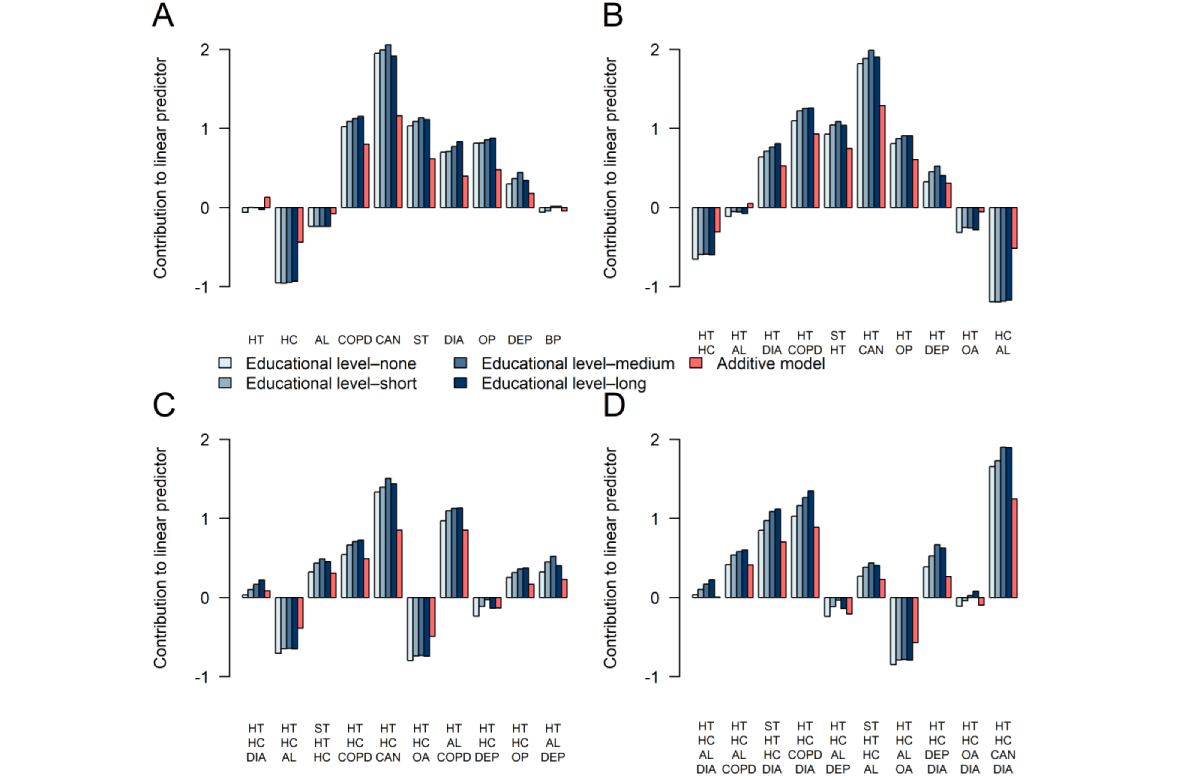
Effects of the 10 most frequent disease portfolio dyads (A), triads (B), tetrads (C), and pentads (D). Effects are shown for male individuals of varying educational attainment levels at the log-hazard rate scale. Comparisons are made to a male individual of the corresponding educational attainment level who only has heart disease (HD). Effects are presented for the all interactions model (different shades of blue) and the only main effects model (red). All comparisons are made at mean age and calendar time. HD is present in all disease portfolios. AL: allergies; BP: back pain; CAN: cancer; COPD: chronic obstructive pulmonary disease; DEP: depression; DIA: diabetes; HC: high cholesterol; HT: hypertension; OA: osteoarthritis; OP: osteoporosis; ST: stroke.

**Figure 6:**
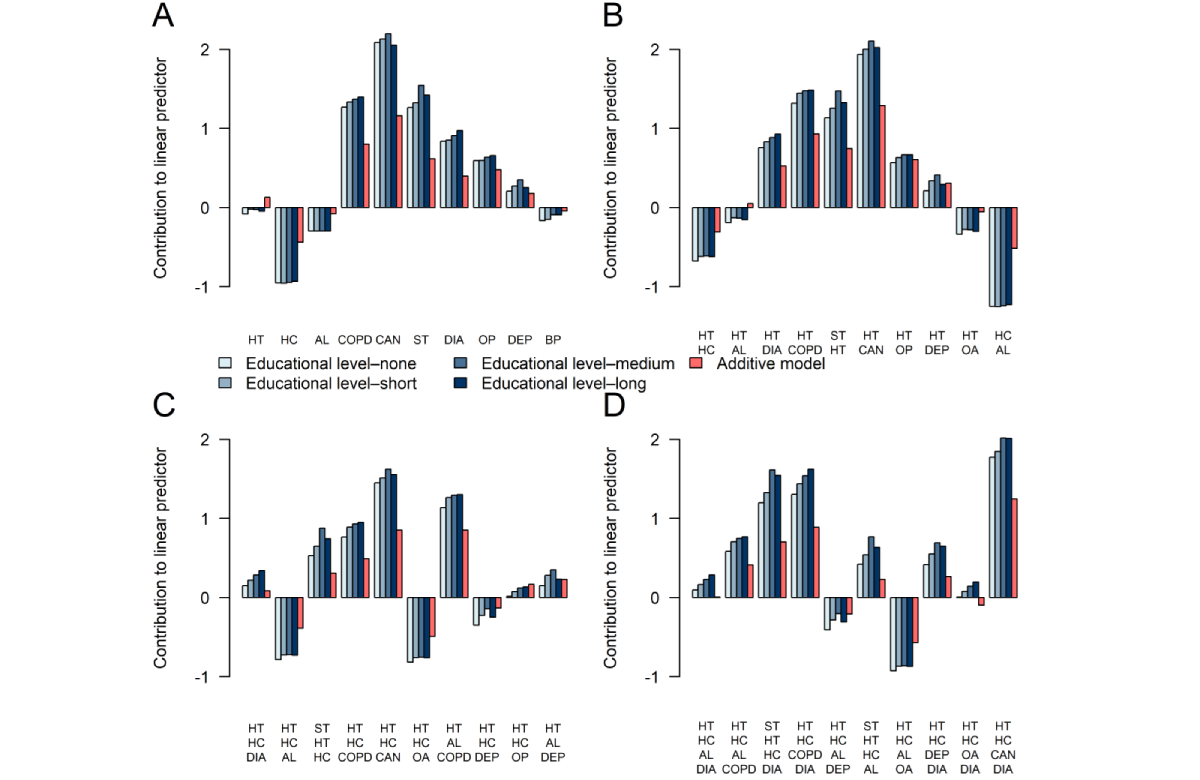
Effects of the 10 most frequent disease portfolio dyads (A), triads (B), tetrads (C), and pentads (D). Effects are shown for female individuals of varying educational attainment levels at the log-hazard rate scale. Comparisons are made to a female individual of the corresponding educational attainment level who only has heart disease (HD). Effects are presented for the all interactions model (different shades of blue) and the only main effects model (red). All comparisons are made at mean age and calendar time. HD is present in all disease portfolios. AL: allergies; BP: back pain; CAN: cancer; COPD: chronic obstructive pulmonary disease; DEP: depression; DIA: diabetes; HC: high cholesterol; HT: hypertension; OA: osteoarthritis; OP: osteoporosis; ST: stroke.

The effects of the 10 most frequent disease portfolio dyads, triads, tetrads, and pentads are presented on the log-hazard scale at increasing educational attainment levels for male individuals in Figure 5 and for female individuals in Figure 6 based on the ALL model. The associated HR estimates are presented in Multimedia Appendices 6 and 7. Disease portfolios including high cholesterol and allergies were of particular concern as many of them had a negative effect, corresponding to a decreased mortality hazard rate relative to an individual diagnosed with HD who was not multimorbid. By comparing effects of the disease portfolios from the ALL model to effects from the OME model, generally, the direction of the effect (positive or negative) agreed between the models for both male and female individuals. However, the magnitude of the effects was greater in the ALL model than in the OME model for almost all disease portfolios, educational attainment levels, and sexes. This indicates an underestimation of the risk associated with a disease portfolio for the positive effects and an overestimation for the negative effects. For some disease portfolios, an inverse social gradient was visible in the educational dimension, where the higher the educational attainment level, the greater the effect of the disease portfolio (refer to, eg, the portfolio [diabetes, hypertension] in Figure 5). Sex-related disparities in disease portfolio effects were also evident. For disease portfolios containing depression and osteoporosis, the effects of the portfolios were greater for male individuals than for female individuals, whereas for COPD, cancer, stroke, and diabetes, the effects were greater for female individuals.

#### Most Complex Disease Portfolios

**Figure 7:**
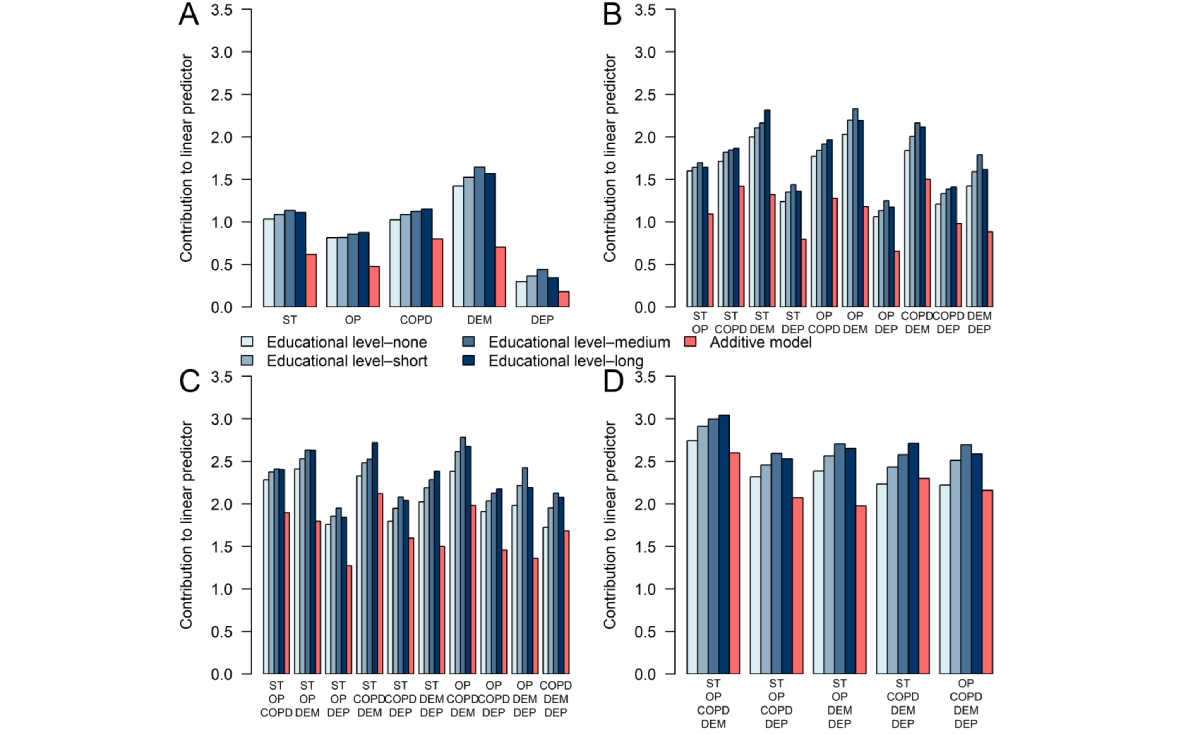
Effects of disease portfolio dyads (A), triads (B), tetrads (C), and pentads (D) involving stroke (ST), osteoporosis (OP), chronic obstructive pulmonary disease (COPD), dementia (DEM), and depression (DEP). Effects are shown for male individuals of varying educational attainment levels at the log-hazard rate scale. Comparisons are made to a male individual of the corresponding educational attainment level who only has heart disease (HD). Effects are presented for the all interactions model (different shades of blue) and the only main effects model (red). All comparisons are made at mean age and calendar time. HD is present in all disease portfolios.

Figure 7 shows the effects of disease portfolios containing combinations of stroke, osteoporosis, COPD, dementia, and depression for male individuals with differing educational attainment levels. These chronic diseases were all part of 5-way interactions, making the effects associated with their portfolios the most complex. For dyads, triads, tetrads, and pentads, the OME model generally yielded lower effects than the ALL model. This implies an underestimation of mortality risk in male individuals for these portfolios when interactions were not modeled. The underestimation was greatest for disease portfolios involving dementia or stroke. Similar results were observed for female individuals but also included a large underestimation of mortality hazard rates for portfolios involving COPD (Multimedia Appendix 8).

#### Disease Portfolios With the Highest Mortality Impact

Table 1 presents the largest HRs for disease portfolio dyads, triads, and tetrads among male and female individuals. Generally, the HRs of the disease portfolios were greater in female individuals; however, the portfolio [schizophrenia] exhibited a greater HR in male individuals. For dyads, the portfolios [cancer], [dementia], [schizophrenia], [stroke], and [COPD] ranked within the top 5 for both sexes. Notably, [cancer] exhibited the largest HR (6.72 for male individuals and 7.59 for female individuals). When considering triads and tetrads, cancer was similarly consistently featured in the top 5 portfolios for both sexes. This indicates that cancer contributes to a greatly increased relative mortality risk whenever present. Among triads, the portfolio [cancer, schizophrenia] had the largest HR for male individuals (13.26) and the second largest for female individuals (13.38). The top-ranking portfolio for female individuals was [cancer, COPD] (HR=15.39), whereas for male individuals, it was the second largest (HR=11.34). Notably, 80% (4/5) of the tetrad portfolios with the highest mortality impact included both cancer and COPD for male and female individuals. As cancer was consistently present in the triads and tetrads with the highest mortality impact, we separately examined the triads and tetrads among portfolios without cancer. The results are presented in Table 2. Upon excluding cancer, we observed that portfolios including dementia and schizophrenia were prominent in most of the triads and tetrads with the highest mortality impact. Among tetrads, the portfolios with the highest mortality impact for male individuals always involved osteoporosis paired with dementia or schizophrenia. In contrast, for female individuals, the tetrads with the highest mortality impact typically consisted of stroke in combination with dementia or schizophrenia.

**Table 1:** The 5 largest hazard ratios (HRs) for dyad, triad, and tetrad disease portfolios.

Rank				Portfolio^a^		HR (99.9% CI)^b^		Individuals^c^, n (%)
**Male** **individuals**								
	**Dyads (n=** **188,910** **)**							
		1	[CAN^d^]		6.72 (6.06-7.45)		6702 (3.55)	
		2	[DEM^e^]		3.99 (3.59-4.43)		1272 (0.67)	
		3	[SCH^f^]		3.04 (2.85-3.24)		888 (0.47)	
		4	[ST^g^]		2.89 (2.66-3.14)		5722 (3.03)	
		5	[COPD^h^]		2.81 (2.55-3.10)		7884 (4.17)	
	**Triads (n=** **229,552** **)**							
		1	[CAN, SCH]		13.26 (11.50-15.29)		66 (0.03)	
		2	[CAN, COPD]		11.34 (9.89-12.99)		1356 (0.59)	
		3	[CAN, OP^i^]		10.35 (9.01-11.90)		433 (0.19)	
		4	[CAN, DEM]		10.06 (8.38-12.07)		131 (0.06)	
		5	[CAN, ST]		9.87 (8.59-11.35)		773 (0.34)	
	**Tetrads (n=** **195,248** **)**							
		1	[CAN, COPD, SCH]		19.21 (16.33-22.60)		28 (0.01)	
		2	[CAN, SCH, ST]		16.82 (14.14-20.01)		14 (0.01)	
		3	[CAN, COPD, OP]		16.40 (14.10-19.07)		157 (0.08)	
		4	[CAN, COPD, ST]		15.92 (13.29-19.07)		168 (0.09)	
		5	[CAN, COPD, DEM]		14.71 (11.59-18.67)		30 (0.02)	
**Female** **individuals**								
	**D** **yads (n=** **148,395** **)**							
		1	[CAN]		7.59 (6.83-8.43)		3559 (2.4)	
		2	[DEM]		4.41 (3.98-4.89)		1180 (0.8)	
		3	[ST]		3.60 (3.27-3.97)		3386 (2.28)	
		4	[COPD]		3.57 (3.23-3.95)		4335 (2.92)	
		5	[SCH]		2.74 (2.56-2.92)		663 (0.45)	
	**T** **riads (n=** **190,272** **)**							
		1	[CAN, COPD]		15.39 (13.51-17.53)		622 (0.33)	
		2	[CAN, SCH]		13.38 (11.70-15.31)		58 (0.03)	
		3	[CAN, DEM]		12.84 (10.60-15.56)		90 (0.05)	
		4	[CAN, ST]		12.65 (10.91-14.67)		296 (0.16)	
		5	[CAN, DIA^j^]		10.44 (9.24-11.80)		251 (0.13)	
	**Tetrads (n=** **177,755** **)**							
		1	[CAN, COPD, SCH]		24.10 (20.45-28.41)		14 (0.01)	
		2	[CAN, COPD, DEM]		23.13 (17.89-29.91)		13 (0.01)	
		3	[CAN, COPD, ST]		22.80 (18.84-27.59)		54 (0.03)	
		4	[CAN, DEM, ST]		19.14 (15.03-24.37)		20 (0.01)	
		5	[CAN, COPD, OP]		17.57 (15.06-20.48)		168 (0.09)	

^a^All portfolios contain the HD diagnosis.

^b^The reference group comprises male or female individuals with only heart disease (HD). HR estimates were aggregated on the log-hazard scale for male and female individuals across all educational attainment levels using weights corresponding to the number of individuals with each portfolio within that subpopulation. Portfolios with <10 individuals were excluded.

^c^The number of unique male or female individuals who had exactly this combination of diseases at any time during the observation period. Percentages are among all male or female individuals observed with dyads, triads, and tetrads, respectively.

^d^CAN: cancer.

^e^DEM: dementia.

^f^SCH: schizophrenia.

^g^ST: stroke.

^h^COPD: chronic obstructive pulmonary disease.

^i^OP: osteoporosis.

^j^DIA: diabetes.

**Table 2:** The 5 largest hazard ratios (HRs) for dyad, triad, and tetrad disease portfolios excluding portfolios with cancer.

Rank				Portfolio^a^		HR (99.9% CI)^b^		Number of individuals^c^
**Male individuals**								
	**Dyads (n=** **182,208** **)**							
		1	[DEM^d^]		3.99 (3.59-4.43)		1272 (0.7)	
		2	[SCH^e^]		3.04 (2.85-2.24)		888 (0.49)	
		3	[ST^f^]		2.89 (2.66-3.14)		5722 (3.14)	
		4	[COPD^g^]		2.81 (2.55-3.10)		7884 (4.33)	
		5	[OP^h^]		2.47 (2.26-2.69)		2341 (1.28)	
	**Triads (n=** **206,638** **)**							
		1	[DEM, OP]		8.58 (7.49-9.84)		257 (0.12)	
		2	[DEM, ST]		7.54 (6.58-8.65)		380 (0.18)	
		3	[COPD, SCH]		7.37 (6.58-8.24)		177 (0.09)	
		4	[DEM, SCH]		7.12 (6.34-8.00)		228 (0.11)	
		5	[SCH, ST]		6.50 (5.80-7.28)		117 (0.06)	
	**Tetrads (n=** **164,266** **)**							
		1	[DEM, OP, ST]		13.37 (11.32-15.78)		98 (0.06)	
		2	[DEM, OP, SCH]		12.36 (10.46-14.61)		52 (0.03)	
		3	[DEM, DIA^i^, OP]		12.09 (10.21-14.31)		19 (0.01)	
		4	[COPD, DEM, OP]		11.90 (10.00-14.16)		42 (0.03)	
		5	[COPD, OP, SCH]		11.72 (10.26-13.40)		26 (0.02)	
**Female individuals**								
	**Dyads (n=** **144,836** **)**							
		1	[DEM]		4.41 (3.98-4.89)		1180 (0.81)	
		2	[ST]		3.60 (3.27-3.97)		3386 (2.34)	
		3	[COPD]		3.57 (3.23-3.95)		4335 (2.99)	
		4	[SCH]		2.74 (2.56-2.92)		663 (0.46)	
		5	[DIA]		2.31 (2.18-2.44)		2939 (2.03)	
	**Triads (n=** **174,861** **)**							
		1	[ST, DEM]		9.77 (8.49-11.24)		268 (0.15)	
		2	[COPD, DEM]		8.68 (7.36-10.24)		113 (0.06)	
		3	[COPD, SCH]		8.44 (7.52-9.47)		106 (0.06)	
		4	[ST, COPD]		8.42 (7.27-9.75)		324 (0.19)	
		5	[OP, DEM]		7.96 (6.99-9.06)		649 (0.37)	
	**Tetrads (n=** **154,975** **)**							
		1	[COPD, DEM, ST]		16.65 (13.54-20.47)		32 (0.02)	
		2	[DEM, DIA, ST]		15.04 (12.95-17.46)		35 (0.02)	
		3	[COPD, SCH, ST]		14.79 (12.56-17.41)		12 (0.01)	
		4	[DEM, OP, ST]		14.58 (12.36-17.20)		142 (0.09)	
		5	[COPD, DEM, OP]		13.86 (11.51-16.70)		72 (0.05)	

^a^All portfolios contain the HD diagnosis.

^b^The reference group comprises male or female individuals with only heart disease (HD). HR estimates were aggregated on the log-hazard scale for male and female individuals across all educational attainment levels using weights corresponding to the number of individuals with each portfolio within that subpopulation. Portfolios with <10 individuals were excluded.

^c^The number of unique male or female individuals who had exactly this combination of diseases at any time during the observation period. Percentages are among all male or female individuals observed with dyads, triads, and tetrads, respectively, excluding those with cancer.

^d^DEM: dementia.

^e^SCH: schizophrenia.

^f^ST: stroke.

^g^COPD: chronic obstructive pulmonary disease.

^h^OP: osteoporosis.

^i^DIA: diabetes.

#### Effect of Sex Across Socioeconomic Subpopulations

**Figure 8:**
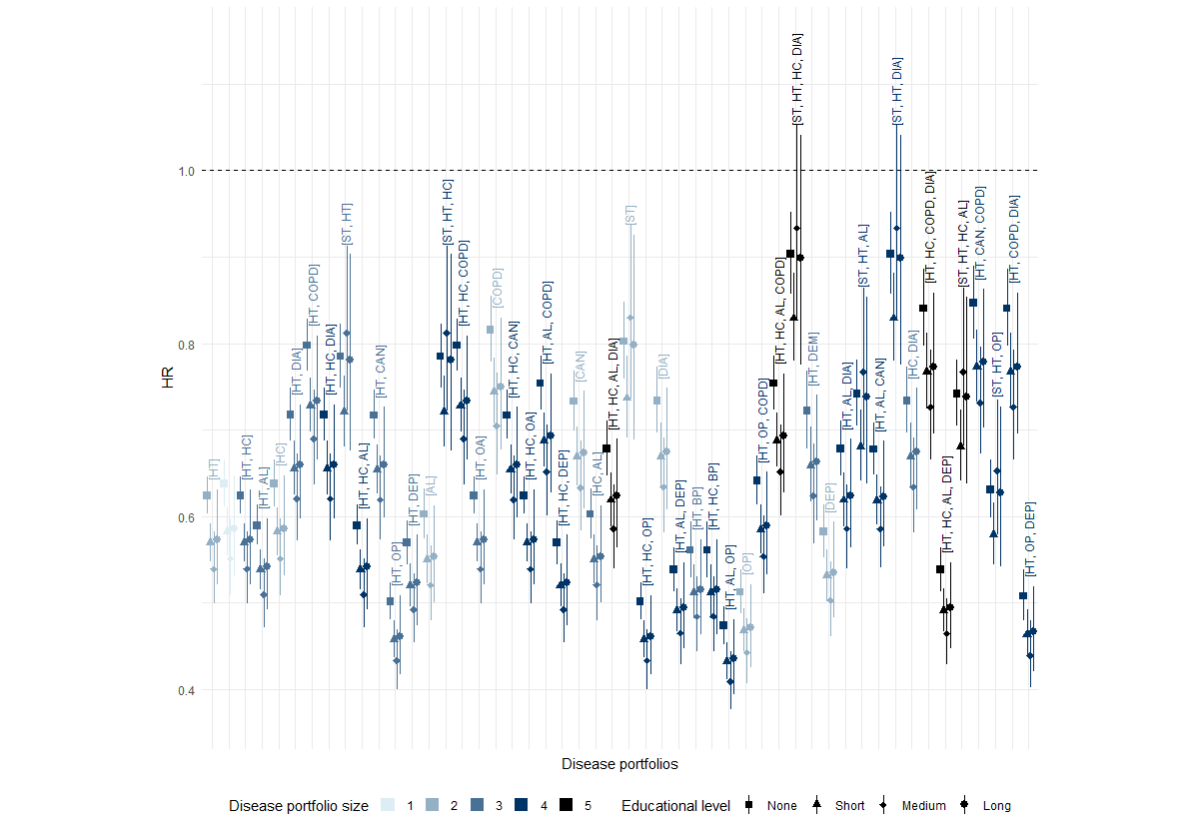
Hazard ratios (HRs) of female (vs male) sex by disease portfolio and educational attainment level. Estimates for the 50 most common disease portfolios are shown with 99.9% CIs. The estimates are presented for each of the educational attainment levels: none, short, medium, and long, indicated by different shapes and always in ascending order from none to long. The reference group comprises male individuals with the same disease portfolio and educational attainment level. The disease portfolios are ordered by prevalence from left to right, with [hypertension (HT)] being the most frequent disease portfolio. All portfolios contain the heart disease (HD) condition, so it is not labeled in the plot. Therefore, the disease portfolio without a label in the plot (the second from the left) corresponds to the disease portfolio with only HD. AL: allergies; BP: back pain; CAN: cancer; COPD: chronic obstructive pulmonary disease; DEM: dementia; DEP: depression; DIA: diabetes; HC: high cholesterol; OA: osteoarthritis; OP: osteoporosis; ST: stroke.

The complex interactions at play indicate that the effect of sex on mortality varies by disease portfolio. This is illustrated in Figure 8, which presents HRs comparing female to male individuals across the 50 most prevalent disease portfolios at different educational levels. Overall, the figure shows a decrease in female mortality risk compared to male mortality risk, with most HRs falling below 1, ranging from 0.41 ([hypertension, allergies, osteoporosis]) to 0.93 ([stroke, high cholesterol, diabetes] and [stroke, hypertension, high cholesterol, diabetes]). However, the magnitude of this decrease varied across comorbidity patterns. For example, portfolios that included osteoporosis consistently showed HRs of <0.66, indicating a notably lower mortality risk for female individuals with these portfolios than for male individuals. Conversely, more complex disease portfolios that included stroke and diabetes—such as [stroke, hypertension, high cholesterol, diabetes] and [stroke, hypertension, diabetes]—had HRs closer to 1, suggesting only a slight reduction in female mortality hazard rate compared to male mortality hazard rate.

#### The Impact of COPD

To illustrate that the effect of a single disease varies depending on the other diseases present in the portfolio, we estimated the effect of COPD in each observed disease portfolio in the population. The aggregated results are shown in Table 3 for male and female individuals of increasing disease portfolio size. The effect of COPD was greatest in triads (HR=2.81 for male individuals and 3.57 for female individuals) and generally higher in female than in male individuals. For increasing disease portfolio sizes, the aggregated effect of COPD decreased considerably with increasing disease portfolio sizes.

**Table 3:** Effect of chronic obstructive pulmonary disease (COPD) for increasing disease portfolio sizes. Each cell is the aggregated effect of COPD (ie, hazard ratio [HR] comparing the portfolio with and without COPD). The effects were aggregated on the log-hazard scale using weights determined based on the occurrence of each specific disease portfolio across the different educational attainment levels for each sex.

Sex	Disease portfolio size						
	2	3	4	5	6	7	8
HR for male individuals	2.81	2.98	2.74	2.50	2.27	2.08	1.91
HR for female individuals	3.57	3.77	3.43	3.08	2.75	2.45	2.19

### Scenarios

**Figure 9:**
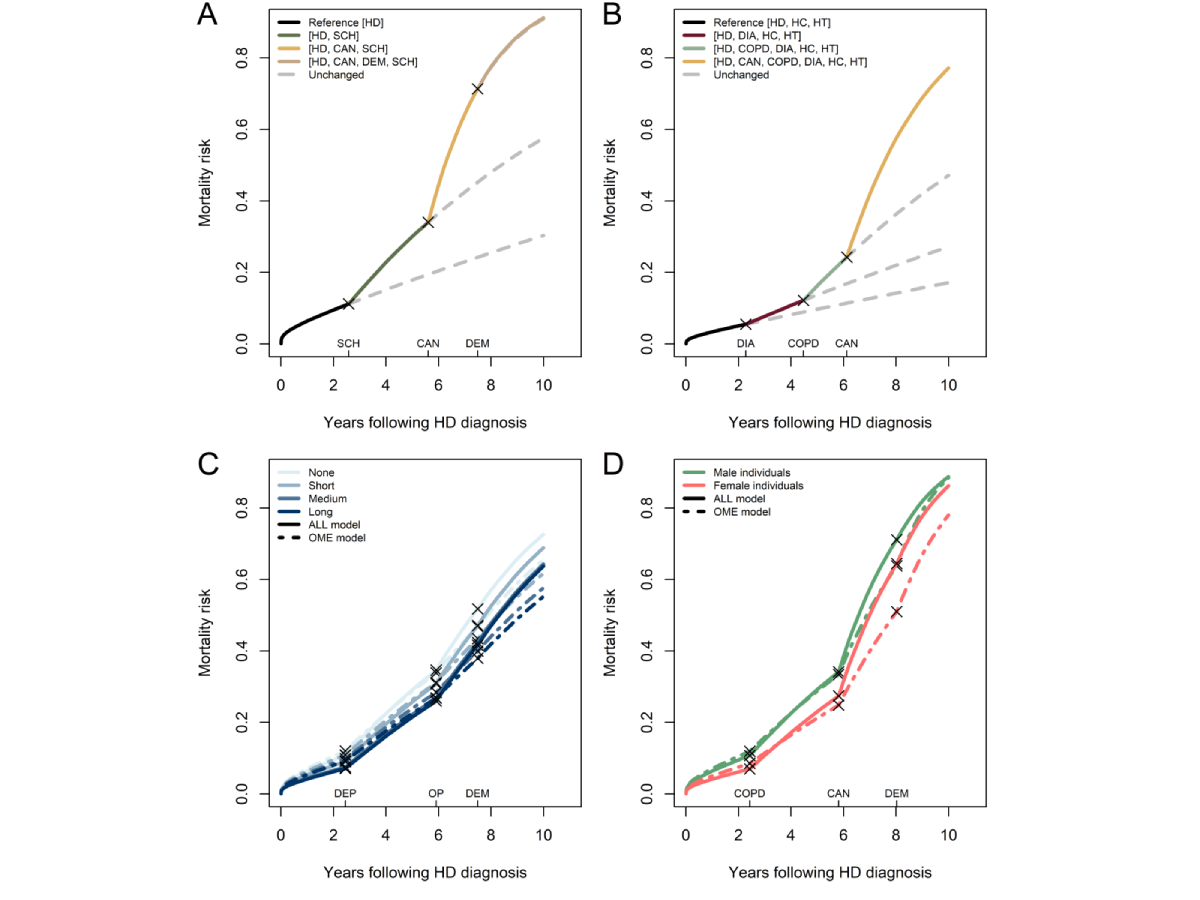
Disease progression scenarios representing the mortality risk over time of a hypothetical (A) male individual with no education at mean age and calendar time who develops schizophrenia (SCH), cancer (CAN), and dementia (DEM) at 2.6, 5.6, and 7.5 years, respectively, following heart disease (HD) diagnosis; (B) male individual with no education who has hypertension (HT), high cholesterol (HC) at time of HD diagnosis and diabetes (DIA), chronic obstructive pulmonary disease (COPD), and CAN at 2.3, 4.8, and 6.1 years, respectively, following HD diagnosis; (C) male individual of varying educational attainment levels who develops depression (DEP), osteoporosis (OP), and DEM at 2.5, 5.9, and 7.5 years, respectively, following HD diagnosis under the all interactions (ALL) model (solid lines) and the additive only main effects (OME) model (dashed lines); and (D) male (green color) and female (red color) individual with no education who develops COPD, CAN, and DEM at 2.4, 5.8, and 8.0 years, respectively, following HD diagnosis under the ALL model (solid lines) and the additive OME model (dashed lines).

We present 4 scenarios in Figure 9 to illustrate how the ALL model’s estimates translate to the risk scale. In Figure 9A, we show the first scenario, which consists of the trajectory of schizophrenia followed by cancer and then dementia. The figure illustrates an increase in the mortality rate with the additions of schizophrenia and cancer to the disease portfolio. However, when dementia diagnosis is obtained, its involvement in interactions prevents a substantial increase in the mortality rate compared to simply continuing undiagnosed. This is despite dementia being the disease with the second-highest mortality impact when considered in isolation (HR=3.99 for male individuals and 4.41 for female individuals; Table 1). The interaction effects between the diseases in the portfolio and dementia create a situation in which adding dementia does not further elevate the mortality hazard rate substantially.

Figure 9B shows a scenario that could resemble the disease trajectory of a male heavy smoker. In this scenario, the patient initially obtains HD diagnosis while also having hypertension and high cholesterol. Over the following years, the patient receives a diabetes diagnosis, which further elevates the mortality risk. The risk accelerates even more with the addition of a COPD diagnosis and, finally, a cancer diagnosis. In Figure 9C, a scenario showing the risk over time for a depression, osteoporosis, and dementia trajectory at different educational attainment levels for both the ALL and OME model is presented. A deviation between the ALL and OME models is most visible at the dementia disease, after which the risk in the ALL model accelerates compared to that in the OME model. In addition, the scenario visualizes that, despite the inverse social gradient of the disease portfolios on the log-hazard scale (Figure 7), lower educational attainment is still associated with a greater risk of death. Another scenario illustrating this relationship is presented in Figure 9D for a COPD, cancer, and dementia trajectory. In this scenario, we observe general increased mortality in male individuals compared to female individuals. However, due to the HRs of the disease portfolios being greater in female compared to male individuals (Table 1), the sex difference decreases over time.

## Discussion

### Principal Findings

Patients with HD will often be diagnosed with other chronic diseases during their lifetime [2,5]. The effect of these co-occurring diseases on adverse outcomes is an important research focus as it is a clinically emerging challenge. In this study on the effect of disease portfolios on time until death, an extended Cox model allowing for time-varying covariates was applied to a large, longitudinal dataset encompassing all Danish adult patients with HD in the period from 1995 to 2015. We identified interactions through a model and data-driven variable selection procedure, revealing the severe diseases depression, stroke, COPD, dementia, and osteoporosis as involved in the most complex interactions. In addition, we estimated a simpler additive model consisting solely of main effects, which, on average, underestimated the effect of severe disease portfolios by a factor of 1.4. We did this to elucidate the importance of considering interaction effects when modeling the mortality risk associated with multiple chronic diseases. To the best of our knowledge, our work is the most extensive study examining the effect of co-occurring diseases on mortality among patients with HD.

We found that depression, stroke, COPD, dementia, and osteoporosis were involved in interaction relationships of the highest order, indicating that, when any of these diseases is added to the disease portfolio of the patient with HD, its risk contribution extensively depends on the other diseases already in the portfolio or the intrinsic variables describing the patient. These diseases were also identified under alternative variable selection procedures. Our comparisons between the interaction model and the simpler additive model showed differences in the magnitude of the effects for several disease portfolios. Overall, if interactions are not modeled, the average effect of disease portfolios on time until death appears underestimated for disease portfolios with >3 diseases (up to a factor of 1.4; Figure 4). For female individuals, this average underestimation also applied to disease portfolios of size 3. We observed an inverse socioeconomic gradient in the educational dimension for some of the most frequent and complex disease portfolios, where the greater the educational attainment level, the greater the associated HR of the disease portfolio (Figures 5-7; Multimedia Appendix 8). We found that cancer was present in all cases in the disease portfolios with the highest mortality impact (Table 1). When considering disease portfolios with the highest mortality impact that did not include cancer, we observed that the psychiatric diseases schizophrenia and dementia frequently appeared in conjunction with osteoporosis for male individuals and in conjunction with stroke for female individuals (Table 2). Schizophrenia also often appeared with cancer among the disease portfolios with the highest mortality impact. These results highlight effect modification when multiple diseases co-occur in the patient with HD, and therefore, interventions should carefully evaluate the entire disease portfolio of the patient with HD.

### Effects and Interactions

The high complexity of the estimated interaction model is clearly illustrated in Figure 3. The figure shows the many dynamics between diseases at play in the HD population, where multiple chronic diseases are rampant. Depression, stroke, COPD, dementia, and osteoporosis were the chronic diseases included in the most complex interactions, also allowing for interactions between these and the patients’ intrinsic factors. When considering interactions between chronic diseases exclusively (the DIO model), we observed that cancer and diabetes were also involved in the most complex interactions (Multimedia Appendix 3). Interactions with the intrinsic variables sex and age might trivially explain some of these interactions involving cancer and diabetes, which could be why they were not identified among the most complex interactions in the ALL model. Nevertheless, most interactions between individual chronic diseases identified in the ALL model variable selection were similarly discovered in either the stable or DIO model variable selections (Multimedia Appendix 5), indicating robustness in the detected interactions.

The consequences of modeling effects of interactions are meticulously visualized on the risk scale in the scenario illustrated in Figure 9A, where the addition of dementia does not change the risk profile of the hypothetical patient much as he already has the severe diseases schizophrenia and cancer along with HD. In fact, many of the effect modifications implied by the presence of interactions led to an attenuation of the combined effect of the diseases compared to their effects in an additive model. Biologically, this is reasonable as the considered patients are generally frail due to their HD, thereby causing the continued addition of chronic diseases to increase frailty before death eventually occurs. Our results showing the effect of COPD decreasing for increasing disease portfolio sizes support this finding (Table 3).

Our analysis showed that both the psychiatric diseases dementia and long-term depression were involved in the most complex interactions (Figure 3). Although not part of 5-way interactions, schizophrenia was involved in 4-way interactions with several other diseases. These high-order interaction effects in disease portfolios with psychiatric diseases complicate the interpretation of their impact on mortality as the effects of having these psychiatric diseases depend heavily on the other chronic diseases present in the portfolio, as well as on intrinsic factors such as age, sex, and socioeconomic position. From a biological point of view, this illustrates the interplay between somatic and psychiatric diseases concerning mortality [37,38]. Studies report increased prevalence and risk of psychiatric diagnoses for patients with cardiovascular diseases and their risk factors [39], and efforts should be made to improve these patients’ psychological function. In addition, several studies indicate an increased mortality risk in psychiatric patients when comorbidities are present [7,37,38]. Indeed, we also found that the psychiatric diseases schizophrenia and dementia were present in the disease portfolios with the highest HRs (Tables 1 and 2). As a result, this study has substantial implications for the priority of identifying psychiatric manifestations of multimorbidity among patients with HD as mortality risk is heavily modified when these diagnoses are present, at least among the chronic diseases and the population considered in this study.

Cancer was present in all portfolio dyads, triads, and tetrads with the highest HRs (Table 1). This finding is supported by previous studies reporting that most deaths from cardiovascular disease occur in patients diagnosed with breast, bladder, and prostate cancer [40]. However, the cancer diagnosis in our study encapsulated a larger spectrum of cancer conditions. Among the triads and tetrads with the highest mortality impact, cancer was often present with schizophrenia. However, when considering portfolios excluding cancer, dyads with dementia had a higher mortality impact. Previous research shows higher cancer mortality rates in individuals with schizophrenia, often attributed to factors such as nonadherence to treatment, diagnostic overshadowing, and limited collaboration between medicine and psychiatry [41]. For patients with HD, our results highlight these combinations of diseases as having some of the most substantial mortality impacts.

We note that, among the variables identified in higher-order interactions, Figure 7 and Multimedia Appendix 8 show differences in effects when comparing estimates from models with and without interactions. These contrasts emphasize the importance of considering the complete disease portfolio of a patient with HD when assessing risk. Our findings show that, when interactions are not recognized, the model underestimates the effect of severe diseases such as cancer, stroke, and COPD while overestimating the effect of less severe diseases such as high cholesterol and allergies (Figure 5). A previous study demonstrated the adverse impact of ignoring statistical interactions in epidemiologic studies, showing a potential bias in main effect parameter estimates [33], which could be a reason for these observed differences. As the underestimation of effects asserted itself even for disease portfolios of small size, it could be attributed to the first few manifestations of multimorbidity (ie, the first diseases developed after HD) being more important for survival than later. While the risk continuously increases with the addition of diagnoses, the individual disease effects do not combine additively. As a result, some patients might reach a high risk profile with just a few diagnoses, trivializing the extra effect of obtaining a new diagnosis, as illustrated by the scenario in Figure 9A. The situation illustrated in Figure 9A with the mortality risk not changing with the addition of a (on its own) deadly chronic disease can only be modeled when interactions are allowed. We speculate that the simple additive model breaks down due to situations such as these, compensating the underestimation of the effect of severe diseases with an overestimation of the effect of more common, less severe diseases. While it was observed that, on average, the additive model underestimated the effect of disease portfolios (Figure 4), it is essential to mention that the individual disease portfolio effect differences were aggregated across the HD population.

In this study, we observed an apparent negative effect of the high cholesterol diagnosis, indicating increased survival relative to an individual without the disease. This artifact can be attributed to the phenomenon that some individuals diagnosed with HD who are also diagnosed with high cholesterol are likely being treated with lipid-modifying agents such as statins, which have many beneficial properties such as cholesterol reduction and anti-inflammatory effects [42,43]. Despite having an additional diagnosis, these individuals diagnosed with HD might represent a less frail part of the HD population who might have a higher degree of health literacy, thus being more aware of their conditions and receiving attention from their general practitioners. Another possible explanation is our use of diagnosis time instead of the time of actual disease onset, which was unknown. High cholesterol is a condition in which a considerable amount of time may pass before diagnosis [44], and among those patients with HD who are undiagnosed, some may have the disease but not be undergoing treatment. It is also essential to consider other consequences of multimorbidity. Increased survival relative to an individual without a particular disease may appear beneficial at first glance. However, it is crucial to recognize that an additional chronic disease introduces new challenges, such as new medication management, consultations with general practitioners and specialists, and potential functional impairments. It is essential to remember that increased survival in these cases does not necessarily equate to improved quality of life.

We found a more pronounced effect in disease portfolios including osteoporosis in male individuals compared to female individuals (Figures 5, 6, and 8; Table 1). Notably, despite the generally higher prevalence of osteoporosis in female individuals compared to male individuals, it is well documented that male individuals diagnosed with osteoporosis experience higher mortality rates than their female counterparts [45]. Our study reaffirms this observation within a nationwide HD population.

Our findings revealed an inverse socioeconomic gradient for some disease portfolios, where the isolated effect of disease portfolios generally increased as educational attainment levels rose (Figures 5-7; Multimedia Appendix 8). Thus, the higher educated the patient, the higher the mortality hazard rate of the disease portfolio compared to a person of the same educational level with only HD. It is widely known that individuals with higher levels of education enjoy better overall health and lower mortality hazard rates than people with lower levels of education [46]. Consequently, given that the reference patient with HD who was not multimorbid was generally healthier in the subpopulation with the highest educational attainment, it is plausible that those who do become multimorbid in this subpopulation experience a comparatively higher relative mortality hazard rate. Hence, when interpreting this inverse social gradient, it is important to bear in mind that the HR reflects the increased relative mortality hazard rate associated with having a specific multimorbid disease portfolio compared to only having HD. Importantly, the inverse social gradient does not directly translate to increased mortality with higher educational level on the risk scale, as illustrated in Figure 7C. Social disparities are extensively documented across various aspects of multimorbidity, including prevalence [21], health care use [47], and transitions between disease portfolios [28]. Our results contribute to this by revealing an inverse social gradient concerning the isolated effect of combinations of chronic diseases on mortality within a nationwide HD population.

As clinical practice, such as guidelines, screening, testing, and treatment for chronic diseases, evolved over the period from 1995 to 2015, our analysis was adjusted for calendar time at HD diagnosis. We systematically assessed the influence of calendar time on the most frequently observed disease portfolios. Generally, we observed increased survival for patients diagnosed more recently compared to earlier (of the 100 most common portfolios, n=98, 98%). However, an inverse trend indicating decreased survival over calendar time was observed for a few disease portfolios, particularly for the portfolio [dementia] and, in many cases, when dementia was combined with diabetes or stroke. It is well known that demographic changes have caused an increase in the prevalence of dementia over the years [48], but as the model is conditional on the disease portfolio, an increased prevalence of dementia over time does not in itself explain the result. We currently lack an explanation for this result and plan to further investigate it in future research.

### Interpretations

This study illustrates that the complexity of addressing the effects of multiple chronic conditions in a large, temporal dataset requires consideration of the individual’s complete disease portfolio. The extended Cox model used throughout this work was chosen because it allows for modeling time-varying variables in a survival context. In addition, it has the advantage of making no assumptions regarding the distribution of the survival times (ie, the underlying hazard function is left unspecified [49]). However, a few assumptions were made about the hazard function, namely, the relationship between covariates and the hazard function. By examining Schoenfeld residuals, we found that, in some cases, the proportional hazard assumption was not fully supported [31], meaning that the effects might vary across time. Therefore, it is essential to interpret the presented effects as weighted averages of the true, possibly time-varying effects across the entire observation period [50]. There are previous studies on the effect of multimorbidity on time to death within HD populations [5,7]. However, the analyses conducted in these studies do not acknowledge that a patient’s multimorbidity state is likely to change dynamically through time (ie, that it is time dependent). The differences in prevalence at time *t*=0 and the end of the observation period (Figure 2) in this study illustrate much progression in disease portfolios. Thus, it is essential to consider this when conducting a temporal statistical analysis. When interpreting effects, it is crucial to keep the population in mind. As the study population was selected and followed up on from the time of HD diagnosis, the individuals considered were generally ill compared to, for example, an individual without any chronic diseases. Furthermore, with Denmark being a European welfare state, the population differs from those of many other countries where individuals may have to pay for examinations; thus, the effects might not be directly comparable due to variations in treatment accessibility.

It is crucial to elaborate further on the contrasts associated with the presented effect estimates. The estimates presented compare a patient with HD who is not multimorbid to a patient with HD who is multimorbid with a specific disease portfolio. In the OME model, the effect of comparing, for example, a patient with HD diagnosed with cancer and COPD to a patient with HD who is not multimorbid would be the same as comparing a patient with HD who also has cancer, COPD, and depression to a patient with HD who also has depression. In other words, the effect of a disease combination in an additive model can be interpreted as having the specific combination of diagnoses in the disease portfolio versus not having it. However, in the presence of high-order interactions, the interpretation is only the increased (or decreased) effect comparing an individual with the particular disease portfolio to an individual without it. This is due to the possibility of interactions with other variables, which modify the effects of the disease combination.

The scenarios in Figure 9 were created to illustrate the workings of the extended Cox model by illustrating how the model estimates the mortality risk over time for the hypothetical individuals diagnosed with HD. However, one should be careful in interpreting these scenarios. They cannot be used prognostically to forecast as time points of portfolio expansions are never known at the time of HD diagnosis as that would be conditioning on future events. These scenarios were solely constructed to represent how the model depicts the mortality risk of a “typical” patient with HD over time. The figures help illustrate how the interaction effects on the log-hazard scale relate to the risk of mortality on the probability scale.

For the results presented in this paper, it is essential to emphasize that the effects and interactions uncovered represent associations, not causal relationships. While our results provide valuable insights into the relationships among the chronic diseases, they should be interpreted as observational associations, which can be informative for hypothesis generation and risk assessment for individual portfolios. Furthermore, a considerable group of individuals had missing educational attainment information in this study. In our analyses, we modeled missing values as separate categories. We also estimated the final ALL model under the multiple imputation framework [51], which led to similar results as those presented.

### Strengths and Weaknesses

The main strength of this study is the entire Danish population of individuals diagnosed with HD observed over a long period using register data. Danish register data are generally of high quality and fully representative of the entire Danish population [52]. In addition, the use of algorithmic diagnoses processing both *International Classification of Diseases, 10th Revision,* diagnosis history and Anatomical Therapeutic Chemical medicine history ensured that the HD population covered both the primary and secondary parts of the Danish health care system. However, there are several limitations associated with this study. Given the observational nature of this study, our results do not enable us to draw causal conclusions. In addition, despite the algorithmic diagnoses previously being shown to be reliable [18], a chronic disease’s true onset comes before diagnosis. This is less of a challenge when diagnoses are considered in a cross-sectional study than in a longitudinal setting. Therefore, as time stamps for true disease onsets are not possible, it is crucial to interpret the longitudinal effects associated with a diagnosis in the context of exactly a diagnosis (ie, the detection of the disease), where the individual may have been ill for some time before that.

### Conclusions

In conclusion, we emphasize the importance of considering a patient’s entire disease portfolio when assessing or modeling risk, avoiding oversimplified silo-based generalizations about the effect of individual diseases. This study highlights the importance of modeling interaction effects when chronic diseases co-occur. Omitting these interactions can result in underestimation of the elevated mortality risk associated with multimorbidity in patients with HD. Through our analysis of a comprehensive nationwide longitudinal dataset of 766,596 patients with HD, we identified sex-related and socioeconomic disparities in disease portfolio HRs. Notably, an inverse socioeconomic gradient was systematically observed for the most common and complex disease portfolios, meaning an increased mortality hazard rate with multimorbidity relative to no multimorbidity as educational attainment level increases. However, absolute mortality risk still decreased with increasing educational attainment due to baseline effects of education. Cancer was present in all disease portfolios with the highest mortality impact. Excluding cancer, disease portfolios including psychiatric chronic diseases were of the highest mortality impact. We identified interactions among all considered co-occurring chronic diseases. We found that stroke, osteoporosis, COPD, dementia, and depression were integral components of the most complex interactions of the highest order. When these chronic diseases co-occur in the patient with HD, their contribution to the patient’s risk profile depends on multiple factors, encouraging a holistic view of the patient’s entire disease portfolio along with their demographic and socioeconomic risk factors.

## Appendix

Multimedia Appendix 1 Algorithmic diagnoses. Algorithms used to define the 15 diagnoses.

Multimedia Appendix 2 Prevalence of diagnoses according to sex.

Multimedia Appendix 3 Graphical representation of disease-disease interactions in the disease interactions only model. A ribbon connects chronic diseases that have any significant interaction (*P*<.001) between them. The connection’s width corresponds to the number of individuals diagnosed with HD developing both diseases throughout the observation period. The ribbon’s color represents the highest-order interaction relationship between 2 diseases. The ribbon chart is ordered by number of connections between diseases, starting from allergies with 5 connections all the way to cancer, which interacts with all the additional diseases

Multimedia Appendix 4 Graphical representation of disease-disease interactions in the stable model. A ribbon connects chronic diseases that have any significant interaction (*P*<.001) between them. The connection’s width corresponds to the number of individuals diagnosed with HD developing both diseases throughout the observation period. The ribbon’s color represents the highest-order interaction relationship between 2 diseases. The ribbon chart is ordered by number of connections between diseases, starting from high cholesterol with 1 connection all the way to chronic obstructive pulmonary disease, which interacts with 11 of the additional diseases. Back pain does not interact with any chronic disease in this model.

Multimedia Appendix 5 Diagnosis-diagnosis interactions identified across the all interactions model, the disease interactions only model, and the stable model. A cell in the table indicates under which models arising from the different variable selection procedures an interaction between the row and column condition is identified. Due to symmetry, only half of the table is presented.

Multimedia Appendix 6 Male hazard ratios (HRs) for the 10 most common disease portfolio dyads, triads, tetrads, and pentads. The results are presented for the all interactions model at the 4 educational attainment levels (none, short, medium, and long) and correspond to the situation presented in Figure 5. The reference group comprises male individuals with only heart disease and the corresponding educational attainment level. Results are also presented for the additive only main effects model. In each disease portfolio group, the disease portfolio HR estimates are presented in order of prevalence, with the upper rows being more prevalent than the lower rows.

Multimedia Appendix 7 Female hazard ratios (HRs) for the 10 most common disease portfolio dyads, triads, tetrads, and pentads. The results are presented for the all interactions model at the 4 educational attainment levels (none, short, medium, and long) and correspond to the situation presented in Figure 6. The reference group comprises female individuals with only heart disease and the corresponding educational attainment level. Results are also presented for the additive only main effects model. In each disease portfolio group, the disease portfolio HR estimates are presented in order of prevalence, with the upper rows being more prevalent than the lower rows.

Multimedia Appendix 8 Effects of disease portfolio dyads (A), triads (B), tetrads (C), and pentads (D) involving stroke, osteoporosis, chronic obstructive pulmonary disease, dementia, and depression. Effects are shown for female individuals of varying educational attainment levels at the log-hazard rate scale. Comparisons are made to a female individual of the corresponding educational attainment level who only has heart disease (HD). Effects are presented for the all interactions model (different shades of blue) and the only main effects model (red). All comparisons are made at mean age and calendar time. The HD condition is present in all disease portfolios.

## Abbreviations

COPD: chronic obstructive pulmonary disease
DIO: disease interactions only
HD: heart disease
HR: hazard ratio
ICD-10: International Statistical Classification of Diseases, Tenth Revision
LASSO: least absolute shrinkage and selection operator
OME: only main effects

## Data Availability

The data used in this study are not publicly available as they consist of sensitive, individual-level information in the form of national register data. According to the Danish data protection legislation, the authors are not allowed to share these sensitive data upon request. Instead, the data are available for research purposes upon request to the Danish Health Authority.
