# Supplementary material for: Co-Occurring Diseases and Mortality in Patients With Chronic Heart Disease, Modeling Their Dynamically Expanding Disease Portfolios: Nationwide Register Study
Source: JMIR Cardio. 2025 Apr 25;9:e57749. doi: 10.2196/57749 (PMC12064962; doi:10.2196/57749)
Supplement: Multimedia Appendix 1 [file cardio_v9i1e57749_app1.docx]

Multimedia Appendix 1. Algorithmic diagnoses. Algorithms used to define the 15 diagnoses.

| Chronic condition | ICD-10 from NPR and PCRR | Definition |
| --- | --- | --- |
| Heart disease | I20, I21, I23-I25, I50, I11, I13 | (DIAG)*^a^* and/or (MEDICINE)*^b^* with ATC: C01A,  C01B, C01D, C01E |
| Stroke | G45, G46, I60-I69 | (DIAG)*^a^* |
| Hypertension | I10-I13, I15 | (DIAG)*^a^* and/or (MEDICINE)*^b^* with ATC: C07B, C03A, C03B, C03E, C03X and/or (MEDICINE)*^b^* with ATC: C03C, C03D, C07A, C09 if they do not have contacts with ICD-10 codes I20.0, I21, I25.1, I50 and/or (MEDICINE)*^b^* with ATC: C08 if they do not have contacts with ICD-10 codes I20-25 |
| High cholesterol | E78.0, E78.2, E78.4, E78.5 | (DIAG)*^a^* and/or (MEDICINE)*^b^* with ATC: C10 |
| Allergies | J30.1-J30.9 | (DIAG)*^a^* and/or (MEDICINE)*^b^* with ATC: V01AA11. V01AA02, V01AA03, V01AA05, R01AC, R01AD, R06A, S01G, R01BA52.  Two prescriptions within a period of two years. |
| Joint disease | M05, M06.0, M06.8, M07.0, M07.1, M10.0, M10.9 | (DIAG)*^a^* |
| Osteoporosis | M80-M82 and/or for persons aged 45 years or older S22.0, S22.1, S32.0, S32.7, S32.8, S42.2, S42.4,  S42.7-S42.9, S52.5-S52.9, S62.0,  S62.1, S72 | (DIAG)*^a^* and/or (MEDICINE)*^b^* with ATC: M05B,  G03XC01, H05AA02, H05AA03 |
| Osteoarthritis | M15-M19 | (DIAG)*^a^* |
| Back pain | M40-M54 | (DIAG)*^a^* |
| Cancer | C00-C43, C45-C97 | (DIAG)*^a^* |
| Chronic obstructive  pulmonary disease  (COPD) | J40-J44, J47, J96 | (DIAG)*^a^* All patients aged 35 years or older at contact. And/or (MEDICINE)*^b^* with ATC: V03AN01 if they do not have contacts with ICD-10 codes J45 or J46, also aged 35 years or older at the date of distribution. And/or (SERVICE)*^c^* with lab services 807113 (lung spirometer test), 807121 (lung function test), also aged 35 years or older at the time of service. |
| Schizophrenia | F20-F22, F25, F28, F29, F31 | (DIAG)*^a^* and/or (MEDICINE)*^b^* N05AX13, N05AX12,  N05AH03, N05AX08 |
| Dementia | F00, G30, F01, F02.0, F03.9,  G31.8B, G31.8E, G31.9, G31.0B | (DIAG)*^a^* All patients aged 60 years or older at contact. And/or (MEDICINE)*^b^* with ATC: N06D, also aged 35 years or older at the date of distribution. |
| Long term use  of antidepressants  (Depression) |  | (MEDICINE)*^b^* with ATC: N06A. Note: At least three different prescriptions with at least 2 years between the first and last one. Patients with previous schizophrenia or dementia are excluded. |
| Diabetes | E10-E14, H28.0, H36.0 | (DIAG)*^a^* and/or (MEDICINE)*^b^* with ATC: A10. A10BA02 is excluded for females aged 20-40 years at the date of distribution |

*^a^* DIAG: All patients at any age unless otherwise specified, who had a hospital inpatient or outpatient encounter with one of the ICD-10 codes specified in the column ICD-10 from NPR and PCRR. Primary (A) and secondary (B) diagnoses are considered. Diagnosis timestamp is taken to be at the midway-point of the first encounter fulfilling the requirements.

*^b^* MEDICINE: All patients at any age unless otherwise specified, who had a minimum of two medicine prescriptions from DNPR on two separate days with the stated ATC codes within a period of one year. Diagnosis timestamp is taken to be the date of medicine distribution when the criteria was fulfilled.

*^c^* SERVICE: All patients who had a minimum of two healthcare services from NHSR with either of the stated lab service codes on two separate days within a period of one year. As healthcare services are reported on a weekly basis, diagnosis timestamp was taken to be at the last day of the week.

If a patient fulfills multiple of the criteria DIAG, MEDICINE and SERVICE, the chronic disease diagnosis timestamp is taken to be the first occurring.
