# Supplementary material for: Co-Occurring Diseases and Mortality in Patients With Chronic Heart Disease, Modeling Their Dynamically Expanding Disease Portfolios: Nationwide Register Study
Source: JMIR Cardio. 2025 Apr 25;9:e57749. doi: 10.2196/57749 (PMC12064962; doi:10.2196/57749)
Supplement: Multimedia Appendix 2 [file cardio_v9i1e57749_app2.docx]

Multimedia Appendix 2. Prevalence of diagnoses according to sex.

| Chronic condition | Male | Female | Total |
| --- | --- | --- | --- |
| Prevalence at *t* = 0 (time of HD diagnosis) | | | |
| Heart disease | 100.00 (406792) | 100.00 (359804) | 100.00 (766596) |
| Hypertension | 64.97 (264306) | 74.69 (268721) | 69.53 (533027) |
| High cholesterol | 32.50 (132213) | 23.52 (84640) | 28.29 (216853) |
| Allergies | 14.51 (59013) | 21.16 (76149) | 17.63 (135162) |
| COPD | 11.03 (44873) | 12.08 (43462) | 11.52 (88335) |
| Diabetes | 11.66 (47426) | 10.12 (36401) | 10.93 (83827) |
| Depression | 6.38 (25955) | 12.30 (44255) | 9.16 (70210) |
| Osteoporosis | 4.13 (16800) | 14.81 (53307) | 9.14 (70107) |
| Stroke | 8.81 (35834) | 9.06 (32596) | 8.93 (68430) |
| Cancer | 6.87 (27934) | 7.26 (26122) | 7.05 (54056) |
| Osteoarthritis | 5.70 (23193) | 8.20 (29496) | 6.87 (52689) |
| Back pain | 5.26 (21409) | 6.39 (23005) | 5.79 (44414) |
| Dementia | 1.39 (5665) | 2.58 (9277) | 1.95 (14942) |
| Schizophrenia | 1.34 (5466) | 1.92 (6926) | 1.62 (12392) |
| Joint disease | 1.34 (5455) | 1.55 (5580) | 1.44 (11035) |
| Lifetime prevalence^a^ | | | |
| Heart disease | 100.00 (406792) | 100.00 (359804) | 100.00 (766596) |
| Hypertension | 77.71 (316100) | 85.11 (306224) | 81.18 (622324) |
| High cholesterol | 51.12 (207983) | 37.94 (136502) | 44.94 (344485) |
| Allergies | 25.24 (102687) | 32.99 (118701) | 28.88 (221388) |
| COPD | 25.07 (101975) | 24.32 (87506) | 24.72 (189481) |
| Osteoporosis | 12.11 (49269) | 33.35 (120002) | 22.08 (169271) |
| Stroke | 21.15 (86021) | 22.12 (79606) | 21.61 (165627) |
| Diabetes | 22.74 (92519) | 18.75 (67450) | 20.87 (159969) |
| Depression | 15.87 (64542) | 25.33 (91156) | 20.31 (155698) |
| Cancer | 20.38 (82923) | 17.92 (64485) | 19.23 (147408) |
| Osteoarthritis | 12.63 (51361) | 16.47 (59268) | 14.43 (110629) |
| Back pain | 11.52 (46846) | 13.43 (48332) | 12.42 (95178) |
| Dementia | 6.34 (25806) | 10.25 (36890) | 8.18 (62696) |
| Schizophrenia | 3.56 (14462) | 5.22 (18778) | 4.34 (33240) |
| Joint disease | 4.26 (17330) | 3.73 (13436) | 4.01 (30766) |

Prevalence are reported as % (n).

^a^Lifetime prevalence is to be understood in the sense that the HD diagnosed individual has been observed to obtain the diagnosis in the time period 1995-2015.
