# Supplementary figures and images for: Co-Occurring Diseases and Mortality in Patients With Chronic Heart Disease, Modeling Their Dynamically Expanding Disease Portfolios: Nationwide Register Study
Source: JMIR Cardio. 2025 Apr 25;9:e57749. doi: 10.2196/57749 (PMC12064962; doi:10.2196/57749)

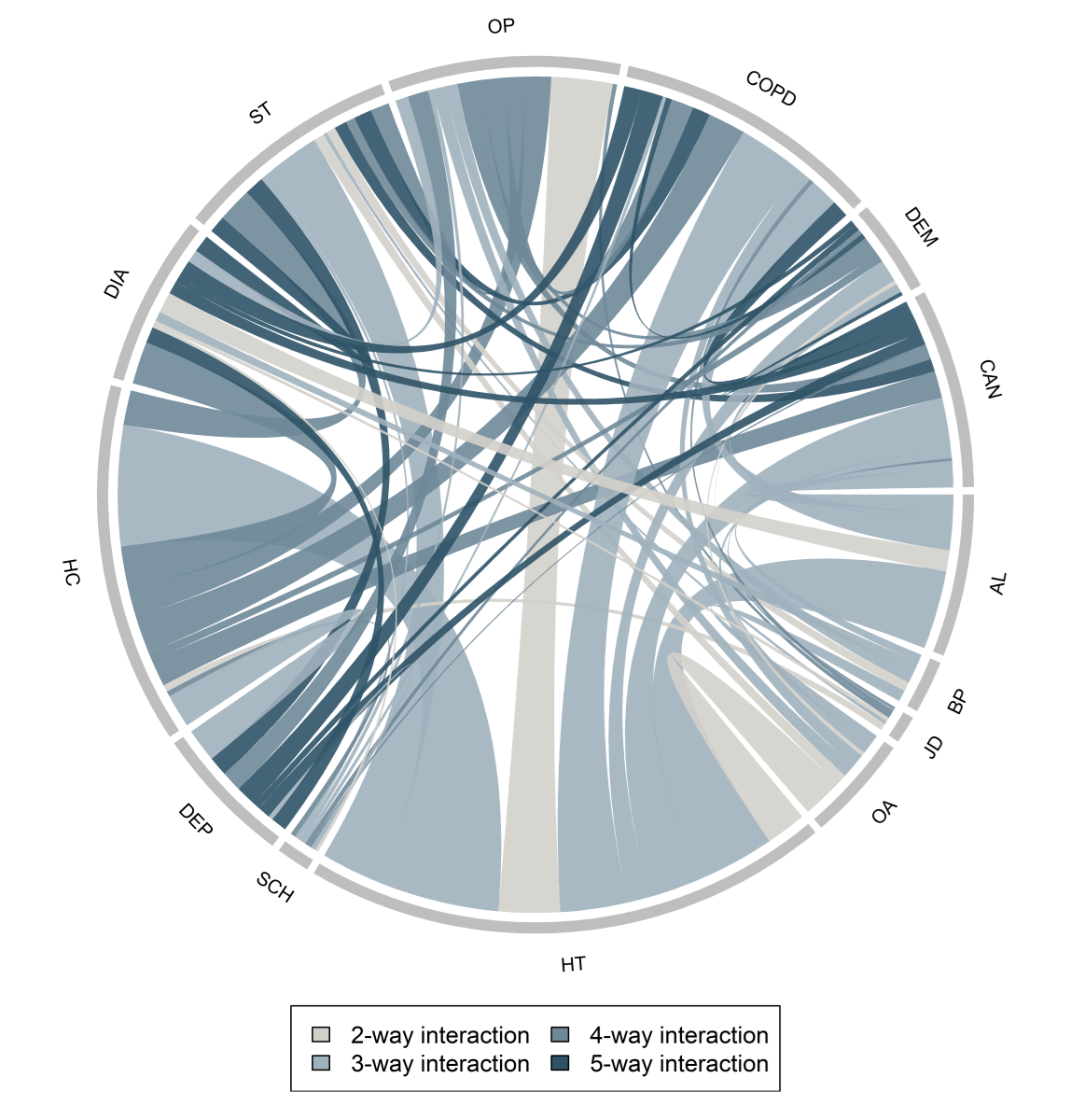

Supplement: Multimedia Appendix 3 [file cardio_v9i1e57749_app3.png]

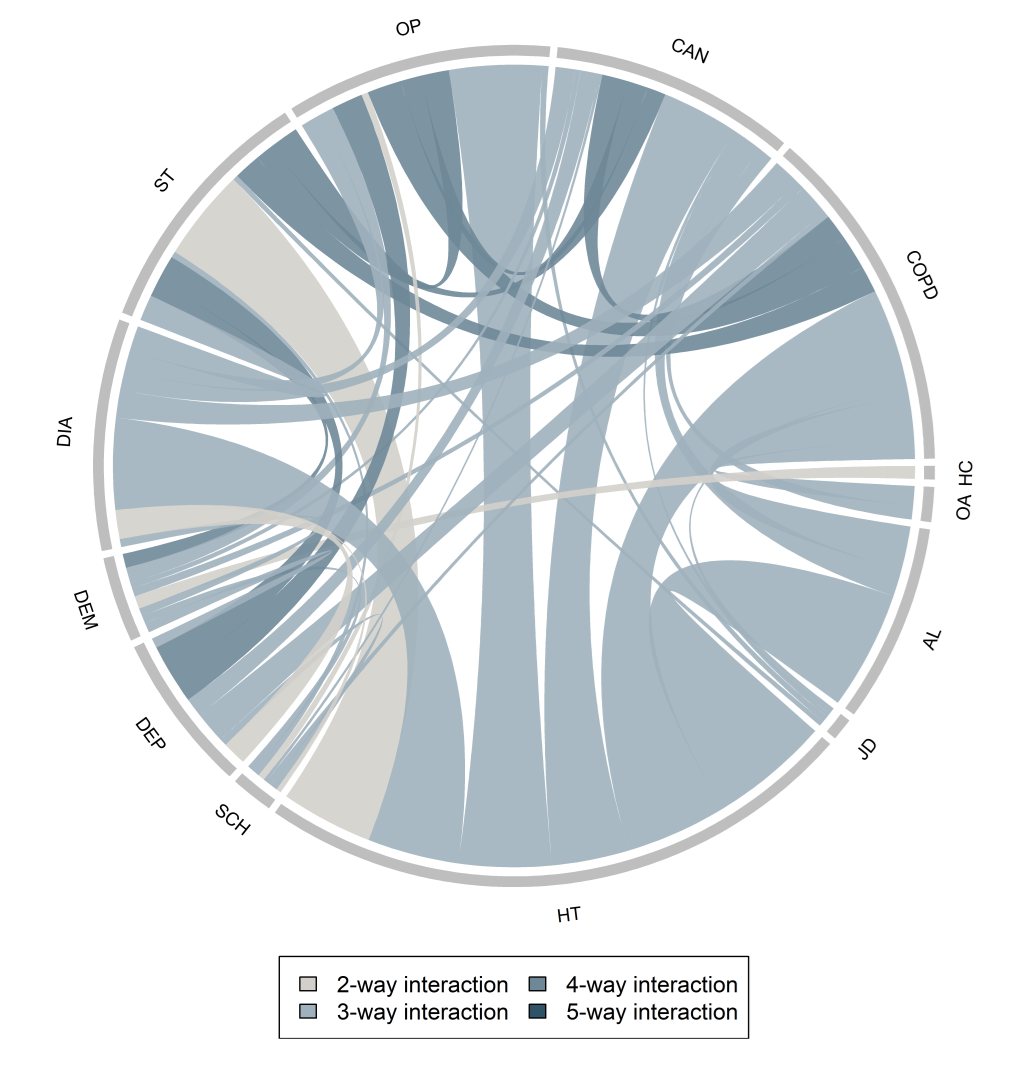

Supplement: Multimedia Appendix 4 [file cardio_v9i1e57749_app4.png]

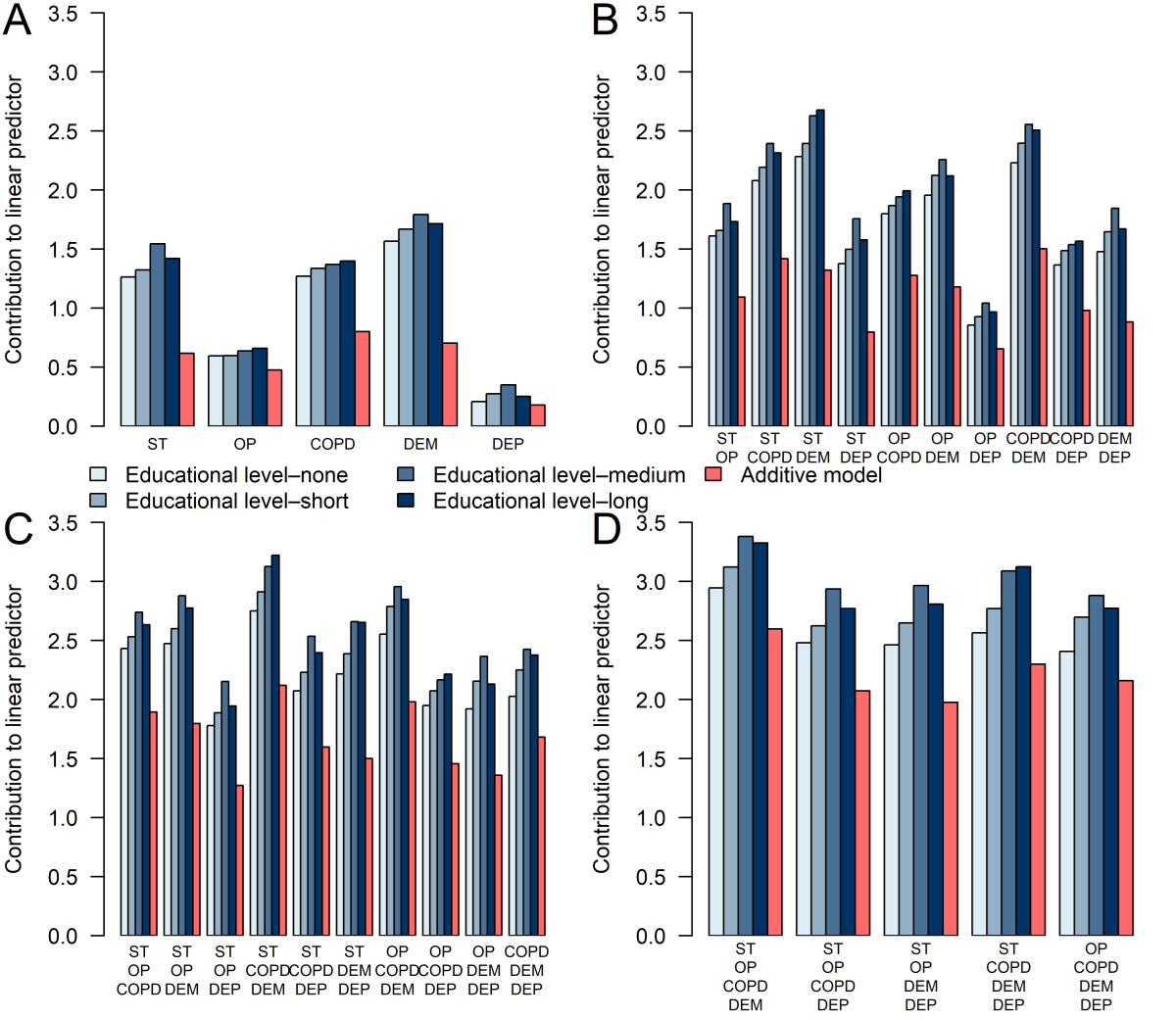

Supplement: Multimedia Appendix 8 [file cardio_v9i1e57749_app8.png]
