# Supplementary material for: Co-Occurring Diseases and Mortality in Patients With Chronic Heart Disease, Modeling Their Dynamically Expanding Disease Portfolios: Nationwide Register Study
Source: JMIR Cardio. 2025 Apr 25;9:e57749. doi: 10.2196/57749 (PMC12064962; doi:10.2196/57749)
Supplement: Multimedia Appendix 5 [file cardio_v9i1e57749_app5.docx]

**Multimedia Appendix 5.** Diagnosis-diagnosis interactions identified across the all interactions model (ALL), the disease interactions only model (DIO), and the stable model (S). A cell in the table indicates under which models arising from the different variable selection procedures an interaction between the row and column condition is identified. Due to symmetry, only half of the table is presented.

| **Chronic condition** | **HT** | **HC** | **AL** | **JD** | **OP** | **OA** | **BP** | **CAN** | **COPD** | **DEM** | **SCH** | **DEP** | **DIA** |
| --- | --- | --- | --- | --- | --- | --- | --- | --- | --- | --- | --- | --- | --- |
|  |  |  |  |  |  |  |  |  |  |  |  |  |  |
| **ST** | ALL, DIO,  S | ALL, DIO |  | ALL, DIO,  S | ALL, DIO,  S | DIO | ALL, DIO | ALL, DIO,  S | ALL, DIO,  S | ALL, DIO,  S | ALL, DIO,  S | ALL, DIO,  S | ALL, DIO,  S |
|  |  |  |  |  |  |  |  |  |  |  |  |  |  |
| **HT** |  | ALL, DIO | ALL, DIO,  S |  | ALL, DIO, S | ALL, DIO |  | ALL, DIO,  S | ALL, DIO,  S | ALL, DIO |  | ALL | S |
|  |  |  |  |  |  |  |  |  |  |  |  |  |  |
| **HC** |  |  |  | ALL, DIO | ALL, DIO | ALL | ALL | ALL, DIO | ALL, DIO | ALL, DIO,  S | ALL, DIO | ALL, DIO | ALL, DIO |
|  |  |  |  |  |  |  |  |  |  |  |  |  |  |
| **AL** |  |  |  |  |  |  |  | ALL, DIO,  S | ALL, DIO,  S | DIO |  | ALL | ALL, DIO |
|  |  |  |  |  |  |  |  |  |  |  |  |  |  |
| **JD** |  |  |  |  | ALL, DIO,  S |  |  | ALL, DIO,  S | ALL, DIO,  S | ALL, DIO |  |  | ALL, DIO |
|  |  |  |  |  |  |  |  |  |  |  |  |  |  |
| **OP** |  |  |  |  |  | ALL, DIO | ALL, DIO | ALL, DIO,  S | ALL, DIO,  S | ALL, DIO,  S | ALL, DIO,  S | ALL, DIO,  S | ALL, DIO,  S |
|  |  |  |  |  |  |  |  |  |  |  |  |  |  |
| **OA** |  |  |  |  |  |  | DIO | ALL, DIO,  S | DIO,  S | ALL, DIO |  |  |  |
|  |  |  |  |  |  |  |  |  |  |  |  |  |  |
| **BP** |  |  |  |  |  |  |  | ALL, DIO |  |  |  |  | ALL, DIO |
|  |  |  |  |  |  |  |  |  |  |  |  |  |  |
| **CAN** |  |  |  |  |  |  |  |  | ALL, DIO,  S | ALL, DIO,  S | ALL, DIO,  S | ALL, DIO,  S | ALL, DIO,  S |
|  |  |  |  |  |  |  |  |  |  |  |  |  |  |
| **COPD** |  |  |  |  |  |  |  |  |  | ALL, DIO,  S | ALL, DIO,  S | ALL, DIO,  S | ALL, DIO,  S |
|  |  |  |  |  |  |  |  |  |  |  |  |  |  |
| **DEM** |  |  |  |  |  |  |  |  |  |  | ALL, DIO,  S | ALL, DIO,  S | ALL, DIO,  S |
|  |  |  |  |  |  |  |  |  |  |  |  |  |  |
| **SCH** |  |  |  |  |  |  |  |  |  |  |  | ALL, DIO,  S | ALL, DIO,  S |
|  |  |  |  |  |  |  |  |  |  |  |  |  |  |
| **DEP** |  |  |  |  |  |  |  |  |  |  |  |  | ALL, DIO,  S |
|  |  |  |  |  |  |  |  |  |  |  |  |  |  |
