# Supplementary material for: Co-Occurring Diseases and Mortality in Patients With Chronic Heart Disease, Modeling Their Dynamically Expanding Disease Portfolios: Nationwide Register Study
Source: JMIR Cardio. 2025 Apr 25;9:e57749. doi: 10.2196/57749 (PMC12064962; doi:10.2196/57749)
Supplement: Multimedia Appendix 6 [file cardio_v9i1e57749_app6.docx]

Multimedia Appendix 6. Male hazard ratios the 10 most common disease portfolio dyads, triads, tetrads and pentads. The results are presented for the ALL model at the four educational attainment levels: None, short, medium and long and correspond to the situation presented in Fig. 5. The reference group comprises males with only HD and the corresponding educational attainment level. Results are also presented for the additive OME model. In each disease portfolio group, the disease portfolio HR estimates are presented in order of prevalence, with the upper rows being more prevalent than the lower rows.
HT = hypertension, HC = high cholesterol, AL = allergies, COPD = chronic obstructive pulmonary disease, CAN = cancer, ST = stroke, DIA = diabetes, OP = osteoporosis, DEP = depression, BP = back pain, OA = osteoarthritis.

| **Dyads** | | | | | | | | | | |
| --- | --- | --- | --- | --- | --- | --- | --- | --- | --- | --- |
|  | **No Education** | | **Short Education** | | **Medium Education** | | **Long Education** | | **Additive model** | |
| **Portfolio***^a^* | **HR** | **CI (99.9%)** | **HR** | **CI (99.9%)** | **HR** | **CI (99.9%)** | **HR** | **CI (99.9%)** | **HR** | **CI (99.9%)** |
| [HT] | 0.94 | (0.90-0.98) | 1.00 | (0.95-1.05) | 1.00 | (0.90-1.10) | 0.97 | (0.88-1.09) | 1.14 | (1.12-1.15) |
| [HC] | 0.39 | (0.36-0.41) | 0.38 | (0.36-0.41) | 0.39 | (0.36-0.42) | 0.39 | (0.36-0.43) | 0.64 | (0.64-0.65) |
| [AL] | 0.79 | (0.75-0.83) | 0.79 | (0.75-0.83) | 0.79 | (0.75-0.83) | 0.79 | (0.75-0.83) | 0.92 | (0.91-0.94) |
| [COPD] | 2.79 | (2.63-2.95) | 2.97 | (2.80-3.16) | 3.08 | (2.77-3.42) | 3.17 | (2.79-3.60) | 2.23 | (2.20-2.25) |
| [CAN] | 7.03 | (6.58-7.50) | 7.34 | (6.83-7.88) | 7.84 | (6.78-9.07) | 6.78 | (5.74-8.02) | 3.19 | (3.15-3.23) |
| [ST] | 2.81 | (2.66-2.97) | 2.97 | (2.80-3.15) | 3.11 | (2.80-3.46) | 3.04 | (2.70-3.41) | 1.85 | (1.83-1.88) |
| [DIA] | 2.01 | (1.93-2.10) | 2.04 | (1.95-2.13) | 2.16 | (2.01-2.33) | 2.30 | (2.11-2.51) | 1.48 | (1.47-1.50) |
| [OP] | 2.25 | (2.12-2.39) | 2.26 | (2.12-2.41) | 2.35 | (2.13-2.60) | 2.40 | (2.13-2.71) | 1.61 | (1.59-1.63) |
| [DEP] | 1.35 | (1.26-1.44) | 1.44 | (1.35-1.54) | 1.55 | (1.38-1.75) | 1.41 | (1.24-1.60) | 1.20 | (1.18-1.21) |
| [BP] | 0.94 | (0.90-0.99) | 0.96 | (0.91-1.01) | 1.01 | (0.92-1.11) | 1.02 | (0.91-1.14) | 0.96 | (0.94-0.97) |
| **Triads** | | | | | | | | | | |
| [HT, HC] | 0.52 | (0.49-0.55) | 0.55 | (0.52-0.58) | 0.55 | (0.50-0.62) | 0.55 | (0.49-0.62) | 0.73 | (0.72-0.75) |
| [HT, AL] | 0.89 | (0.85-0.94) | 0.95 | (0.90-1.00) | 0.95 | (0.85-1.05) | 0.93 | (0.83-1.03) | 1.05 | (1.03-1.07) |
| [HT, DIA] | 1.90 | (1.79-2.01) | 2.04 | (1.91-2.17) | 2.15 | (1.91-2.42) | 2.25 | (1.97-2.56) | 1.69 | (1.66-1.72) |
| [HT, COPD] | 2.99 | (2.83-3.17) | 3.39 | (3.19-3.62) | 3.50 | (3.06-4.00) | 3.53 | (3.02-4.12) | 2.53 | (2.49-2.58) |
| [ST, HT] | 2.53 | (2.37-2.69) | 2.84 | (2.65-3.04) | 2.96 | (2.58-3.40) | 2.83 | (2.43-3.30) | 2.11 | (2.07-2.15) |
| [HT, CAN] | 6.18 | (5.84-6.53) | 6.59 | (6.21-7.00) | 7.29 | (6.51-8.16) | 6.72 | (5.93-7.61) | 3.63 | (3.15-3.23) |
| [HT, OP] | 2.24 | (2.10-2.39) | 2.39 | (2.23-2.57) | 2.48 | (2.16-2.84) | 2.48 | (2.12-2.89) | 1.83 | (1.80-1.87) |
| [HT, DEP] | 1.38 | (1.30-1.48) | 1.57 | (1.46-1.69) | 1.69 | (1.46-1.95) | 1.50 | (1.28-1.76) | 1.36 | (1.33-1.39) |
| [HT, OA] | 0.73 | (0.69-0.77) | 0.78 | (0.73-0.82) | 0.77 | (0.70-0.86) | 0.76 | (0.67-0.85) | 0.95 | (0.93-0.97) |
| [HC, AL] | 0.30 | (0.28-0.33) | 0.30 | (0.28-0.33) | 0.31 | (0.28-0.34) | 0.31 | (0.28-0.34) | 0.60 | (0.59-0.61) |
| **Tetrads** | | | | | | | | | | |
| [HT, HC, DIA] | 1.03 | (0.98-1.09) | 1.11 | (1.04-1.18) | 1.18 | (1.05-1.33) | 1.25 | (1.09-1.43) | 1.09 | (1.07-1.11) |
| [HT, HC, AL] | 0.49 | (0.47-0.52) | 0.52 | (0.49-0.56) | 0.53 | (0.47-0.59) | 0.52 | (0.46-0.59) | 0.68 | (0.66-0.69) |
| [ST, HT, HC] | 1.38 | (1.29-1.47) | 1.54 | (1.44-1.66) | 1.63 | (1.41-1.88) | 1.58 | (1.34-1.85) | 1.36 | (1.33-1.39) |
| [HT, HC, COPD] | 1.72 | (1.62-1.83) | 1.94 | (1.81-2.08) | 2.03 | (1.75-2.34) | 2.07 | (1.75-2.45) | 1.63 | (1.60-1.67) |
| [HT, HC, CAN] | 3.80 | (3.57-4.03) | 4.04 | (3.79-4.31) | 4.51 | (3.99-5.11) | 4.21 | (3.67-4.84) | 2.34 | (2.29-2.40) |
| [HT, HC, OA] | 0.45 | (0.43-0.48) | 0.48 | (0.45-0.51) | 0.48 | (0.43-0.54) | 0.48 | (0.42-0.54) | 0.61 | (0.60-0.63) |
| [HT, AL, COPD] | 2.64 | (2.48-2.80) | 2.99 | (2.80-3.20) | 3.08 | (2.69-3.53) | 3.11 | (2.65-3.64) | 2.34 | (2.29-2.39) |
| [HT, HC, DEP] | 0.79 | (0.74-0.85) | 0.89 | (0.83-0.97) | 0.97 | (0.83-1.13) | 0.87 | (0.74-1.04) | 0.88 | (0.86-0.90) |
| [HT, HC, OP] | 1.29 | (1.20-1.39) | 1.37 | (1.27-1.48) | 1.44 | (1.24-1.66) | 1.45 | (1.23-1.72) | 1.18 | (1.15-1.21) |
| [HT, AL, DEP] | 1.38 | (1.29-1.48) | 1.57 | (1.45-1.69) | 1.68 | (1.45-1.95) | 1.49 | (1.27-1.76) | 1.26 | (1.23-1.29) |
| **Pentads** | | | | | | | | | | |
| [HT, HC, AL, DIA] | 1.03 | (0.97-1.10) | 1.11 | (1.04-1.19) | 1.18 | (1.05-1.34) | 1.25 | (1.09-1.44) | 1.01 | (0.98-1.03) |
| [HT, HC, AL, COPD] | 1.52 | (1.42-1.62) | 1.71 | (1.59-1.84) | 1.79 | (1.54-2.07) | 1.82 | (1.54-2.16) | 1.51 | (1.47-1.55) |
| [ST, HT, HC, DIA] | 2.34 | (2.18-2.51) | 2.65 | (2.45-2.86) | 2.96 | (2.55-3.45) | 3.06 | (2.58-3.62) | 2.02 | (1.97-2.07) |
| [HT, HC, COPD, DIA] | 2.80 | (2.61-3.00) | 3.20 | (2.96-3.45) | 3.54 | (3.03-4.13) | 3.84 | (3.21-4.60) | 2.43 | (2.37-2.49) |
| [HT, HC, AL, DEP] | 0.79 | (0.73-0.85) | 0.89 | (0.82-0.97) | 0.97 | (0.83-1.13) | 0.87 | (0.73-1.04) | 0.81 | (0.79-0.83) |
| [ST, HT, HC, AL] | 1.31 | (1.22-1.40) | 1.47 | (1.36-1.58) | 1.55 | (1.34-1.79) | 1.50 | (1.27-1.76) | 1.26 | (1.23-1.29) |
| [HT, HC, AL, OA] | 0.43 | (0.40-0.46) | 0.45 | (0.42-0.49) | 0.46 | (0.41-0.51) | 0.45 | (0.40-0.52) | 0.57 | (0.55-0.58) |
| [HT, HC, DEP, DIA] | 1.48 | (1.37-1.59) | 1.69 | (1.55-1.84) | 1.95 | (1.66-2.29) | 1.86 | (1.55-2.24) | 1.30 | (1.27-1.34) |
| [HT, HC, OA, DIA] | 0.90 | (0.84-0.96) | 0.96 | (0.89-1.03) | 1.02 | (0.90-1.16) | 1.08 | (0.94-1.25) | 0.91 | (0.88-0.93) |
| [HT, HC, CAN, DIA] | 5.24 | (4.90-5.62) | 5.64 | (5.24-6.07) | 6.69 | (5.84-7.67) | 6.66 | (5.71-7.76) | 3.48 | (3.39-3.56) |

*^a^* All portfolios contain the HD diagnosis.
